# Supplementary material for: Direct generation of protein conformational ensembles via machine learning
Source: Nat Commun. 2023 Feb 11;14:774. doi: 10.1038/s41467-023-36443-x (PMC9922302; doi:10.1038/s41467-023-36443-x)
Supplement: Supplementary file 1 — Supplementary Information [file 41467_2023_36443_MOESM1_ESM.pdf]

Supplementary Information

Direct Generation of Protein Conformational Ensembles via Machine Learning

Giacomo Janson, Gilberto Valdes-Garcia, Lim Heo, and Michael Feig*

Department of Biochemistry and Molecular Biology, Michigan State University, East Lansing, MI 48824, USA

*Corresponding author: mfeiglab@gmail.com

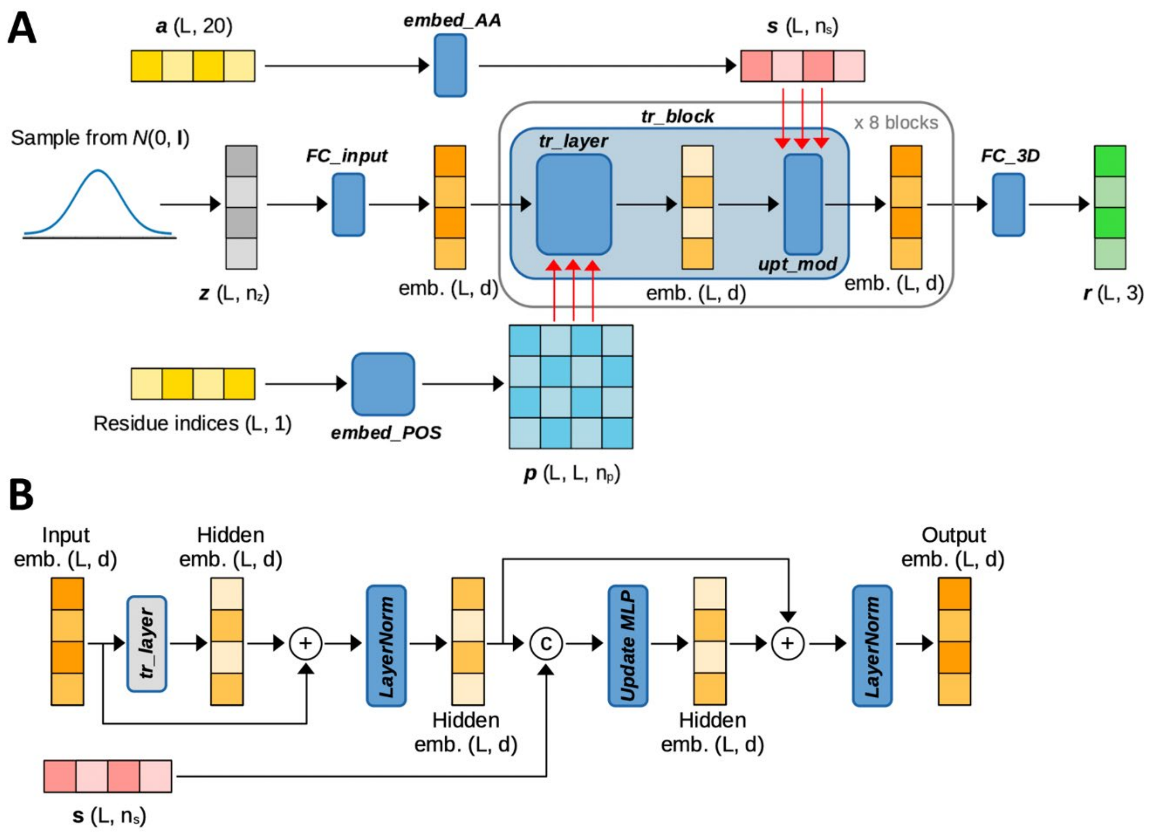

Supplementary Fig. 1. Architecture of the generator network of idpGAN. A: outline of the network. For a protein of length L, a latent tensor $\mathbf{z} \in \mathbb{R}^{L \times n_\mathrm{z}}$, with n_z = 16, is sampled from a Gaussian prior. This tensor is converted in an embedding sequence of L tokens with dimension d = 64 by a fully-connected module acting position-wise (FC_input, **Supplementary Table 4**). The embedding is then processed by a series of n_t= 8 transformer blocks (tr_block). Each block is composed of two sub-modules, a "transformer layer" (tr_layer) and an "updater module" (upt_mod) based on a previous network (see ref. 34 in the main text). A "transformer layer" receives as input an embedding and updates it through a self-attention mechanism (ref. 34 in the main text). We use n_h = 8 attention heads and d_model = 128 as hyper-parameters. Different from the original transformer, each layer uses 2D relative position encoding $\mathbf{p} \in \mathbb{R}^{L \times L \times n_\mathrm{p}}$ (with n_p = 64) as in AF2 (ref. 10 in the main text). The encoding **p** is derived through an embedding module (embed_POS), where each pair of residues in a protein is labeled with a learnable n_p-dimensional vector associated with their sequence separation (i.e., the difference between residue indices). The separation values are clipped from -24 to 24. The same **p** is used in all blocks of the network (triple red arrows in the figure). For each block, we first linearly project **p** onto a bias term for each attention head and then add the biases to the logits values of the corresponding attention maps. The "updater module", shown in panel **B**, is composed of two layer normalization operations and a fully-connected module (update_MLP, **Supplementary Table 4**). In contrast to the original implementation, we do not use dropout, since we found it to negatively impact our GAN performance. An "updater module" also receives as input an encoding $\mathbf{s} \in \mathbb{R}^{L \times n_\mathrm{s}}$ (with n_s = 32). In **s**, each of the 20 amino acid types is associated with a learnable n_s-dimensional vector through an embedding layer (embed_AA) that takes as input a tensor $\mathbf{a} \in \mathbb{R}^{L \times 20}$ storing one hot encodings for amino acid types. To inject **s**, we concatenate it along the feature axis to the tensor produced by the first layer normalization. The same **s** is injected into all blocks of the network (triple red arrows in the figure). The output of the final block is converted in a molecular conformation **r** through a fully-connected module acting position-wise (FC_3D, **Supplementary Table 4**).

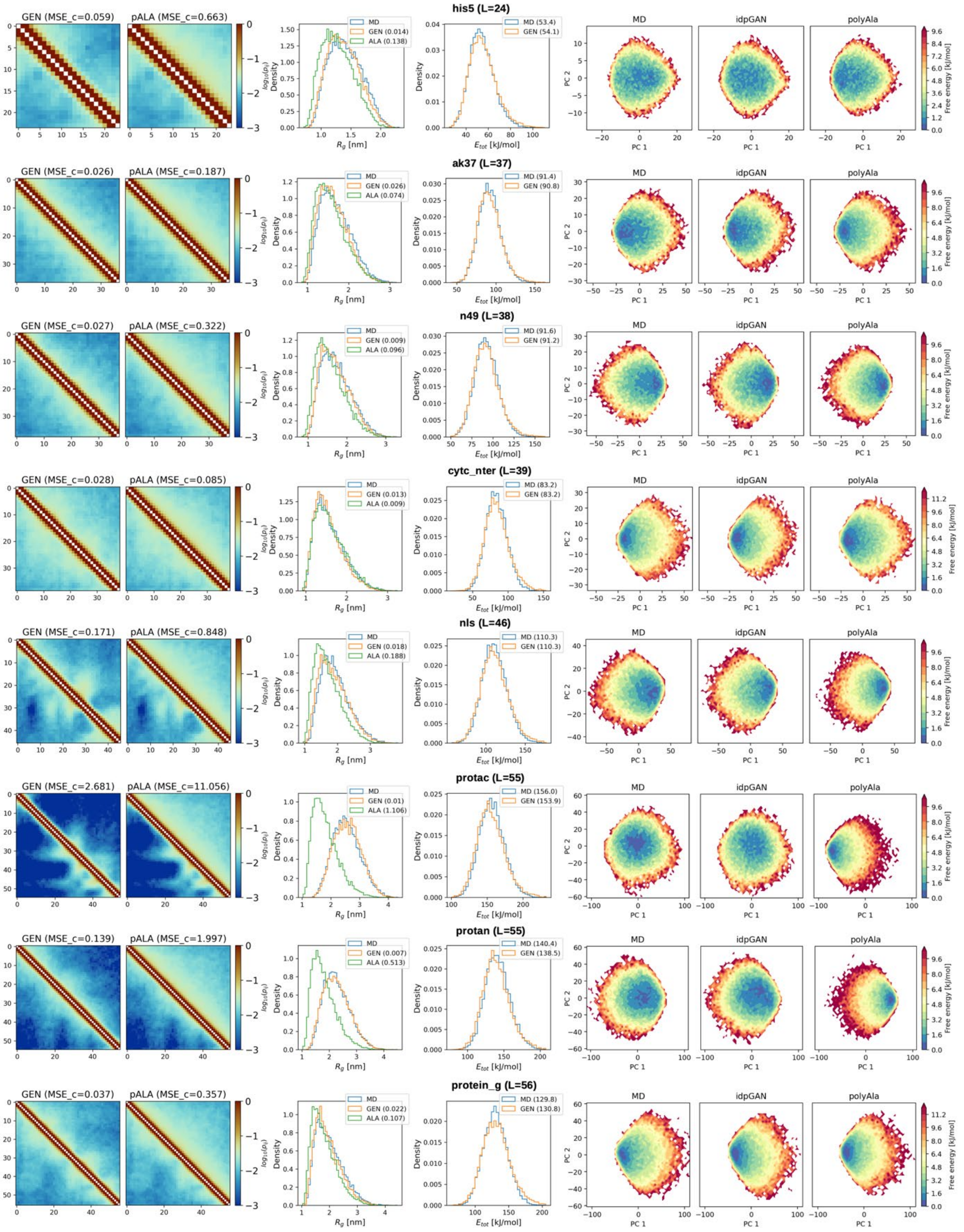

Supplementary Fig. 2. Evaluation of idpGAN on IDP_test proteins. Contact maps, radius-of-gyration distributions, energy distributions and potential of mean force profiles in PCA space from generated (GEN) and polyAla (ALA) ensembles vs. ensembles from MD snapshots for his5, ak37, n49, cytc_nter, nls, protac, protan and protein_g. See **Fig. 2** and **3** for more details.

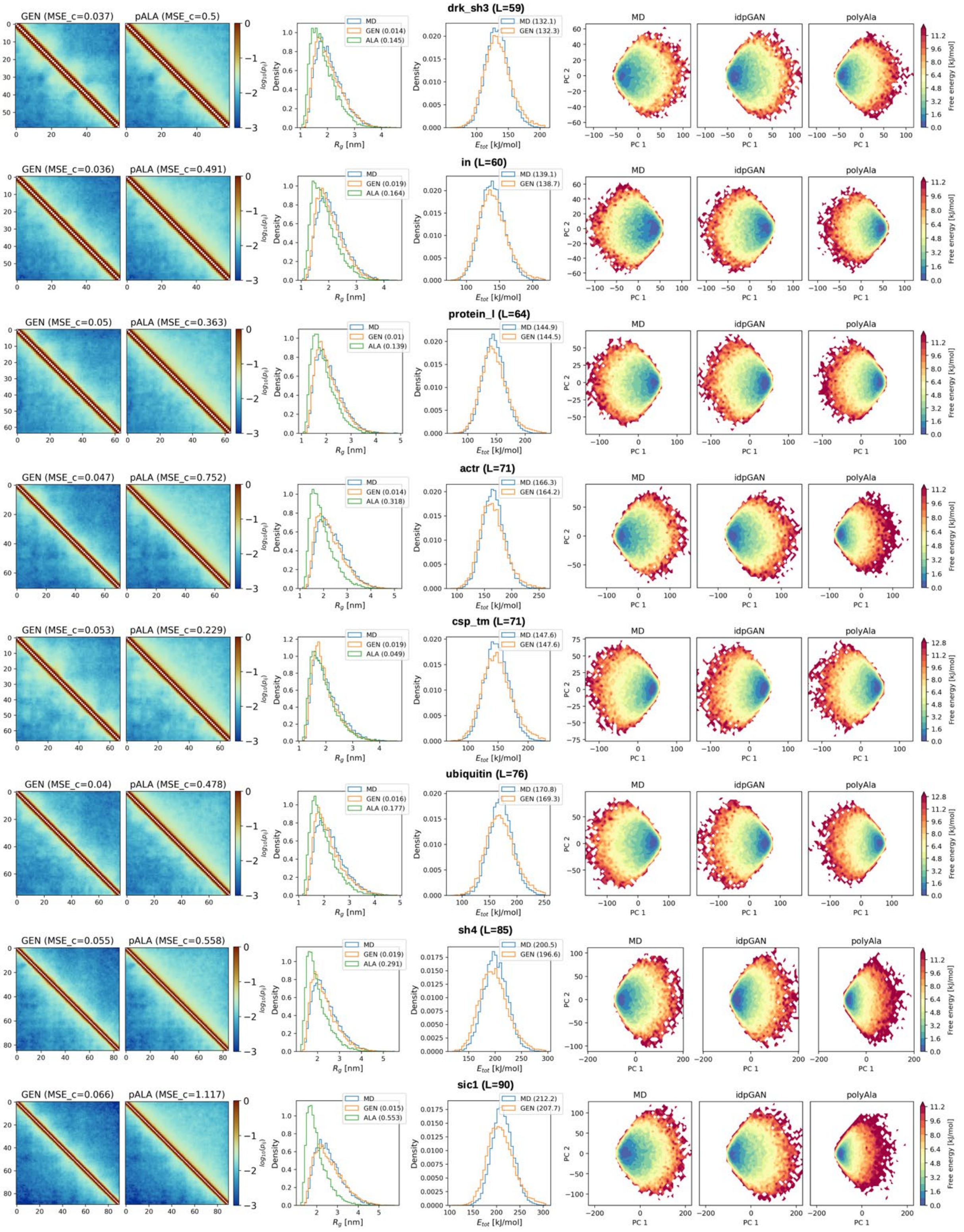

Supplementary Fig. 3. Evaluation of idpGAN on IDP_test proteins. Contact maps, radius-of-gyration distributions, energy distributions and potential of mean force profiles in PCA space from generated (GEN) and polyAla (ALA) ensembles vs. ensembles from MD snapshots for drk_sh3, in, protein_l, actr, csp_tm, ubiquitin, sh4 and sic1. See **Fig. 2** and **3** for more details.

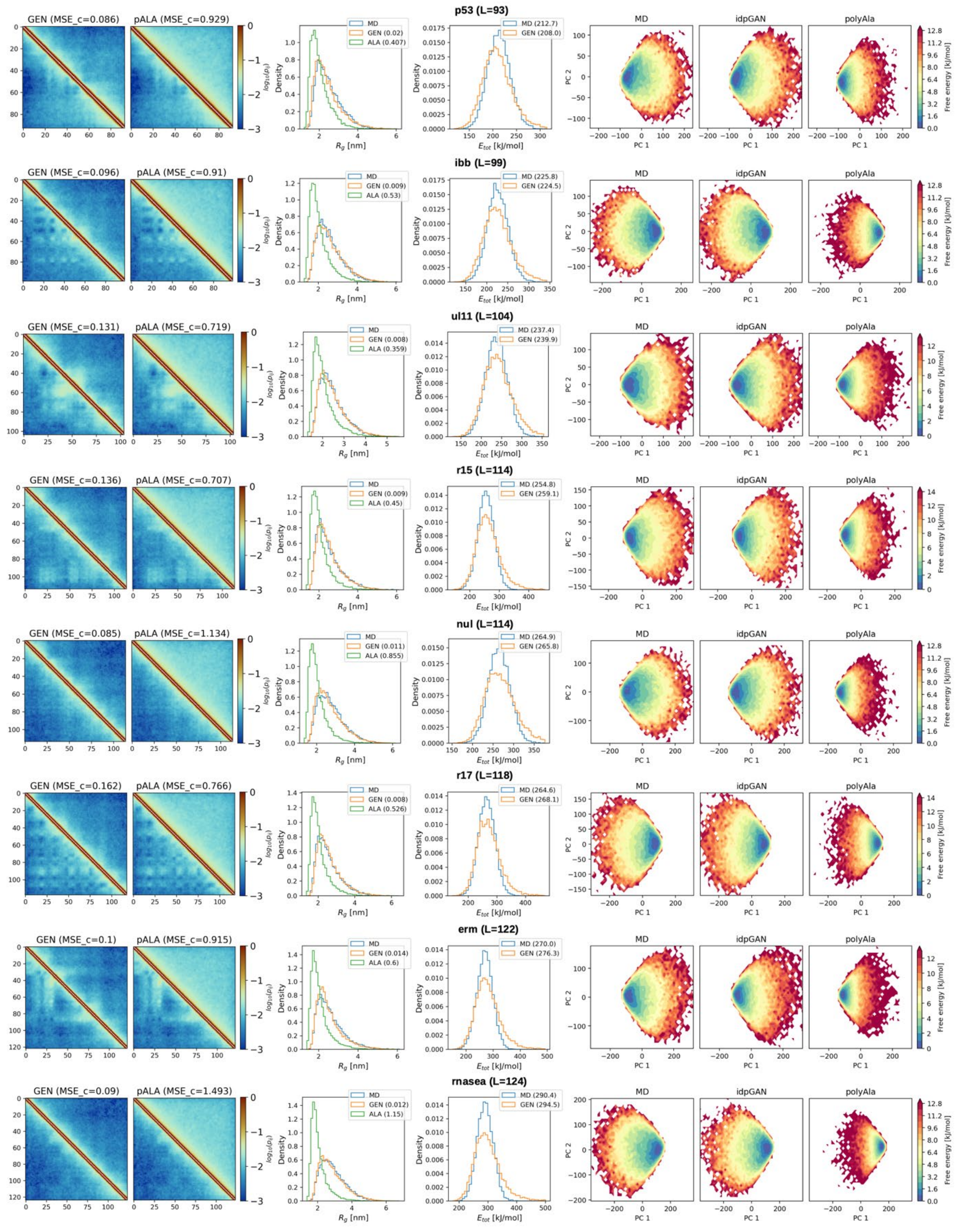

Supplementary Fig. 4. Evaluation of idpGAN on IDP_test proteins. Contact maps, radius-of-gyration distributions, energy distributions and potential of mean force profiles in PCA space from generated (GEN) and polyAla (ALA) ensembles vs. ensembles from MD snapshots for p53, ibb, ul11, r15, nul, r17, erm and rnasea. See **Fig. 2** and **3** for more details.

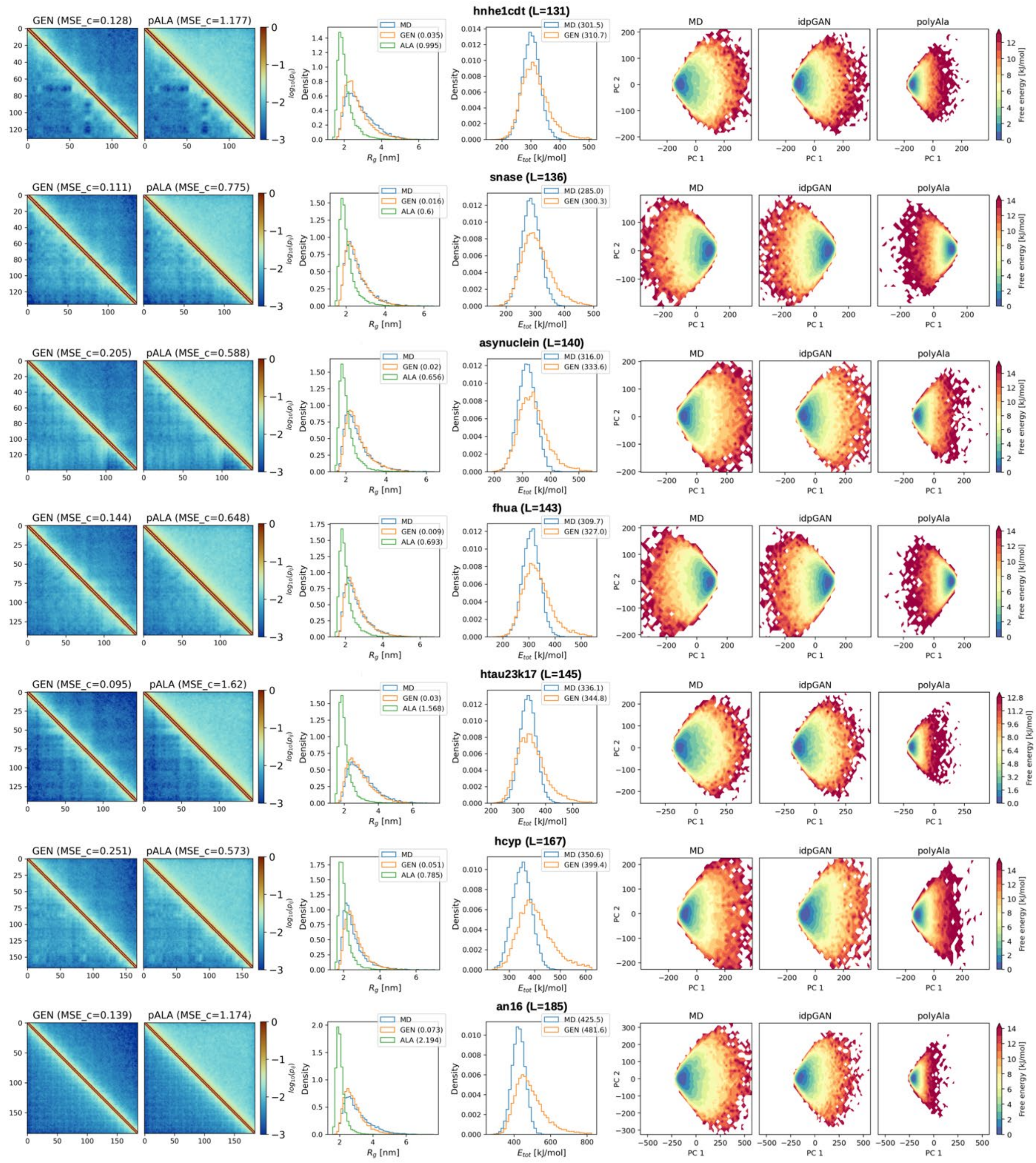

Supplementary Fig. 5. Evaluation of idpGAN on IDP_test proteins. Contact maps, radius-of-gyration distributions, energy distributions and potential of mean force profiles in PCA space from generated (GEN) and polyAla (ALA) ensembles vs. ensembles from MD snapshots for hnhe1cdt, snase, asynuclein, fhua, htau23k17, hcyp and an16. See **Fig. 2** and **Fig. 3** for more details.

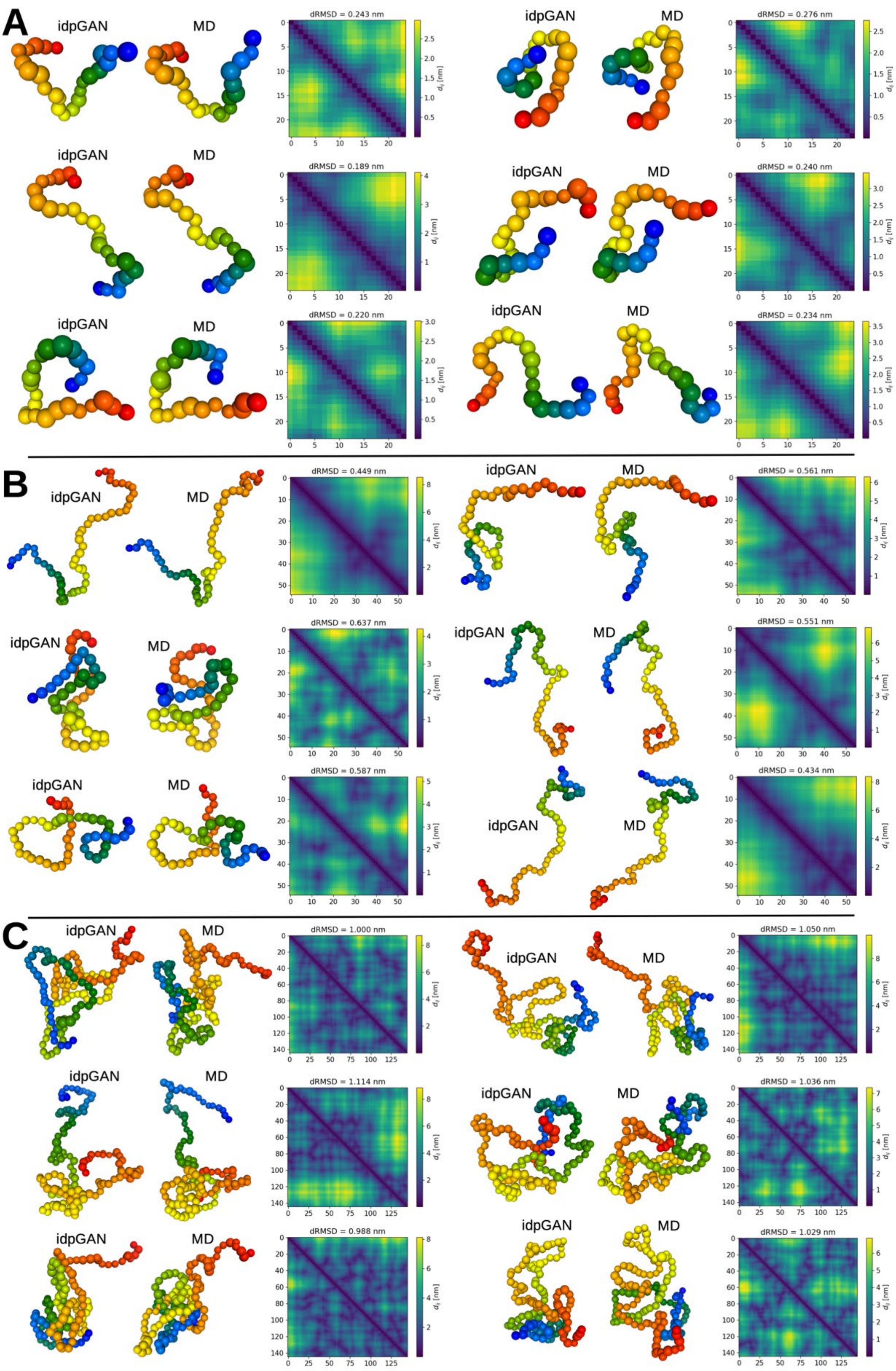

Supplementary Fig. 6. Structures generated by idpGAN. Panels **A** to **C** show six generated snapshots for the his5, protac and htau23k17 proteins of the IDP_test set, respectively. Each snapshot is shown beside its nearest neighbor (in terms of dRMSD) in the MD ensemble of the same protein. Structures are rendered with NGLview. Residues are colored according to their index (red is N-terminal, blue is C-terminal). The distance matrices of the pairs, and their dRMSD value, are also shown (idpGAN in the upper triangles and MD in the lower ones). The generated conformations were not cherry-picked but were randomly extracted from an ensemble of 10,000 conformations for each protein.

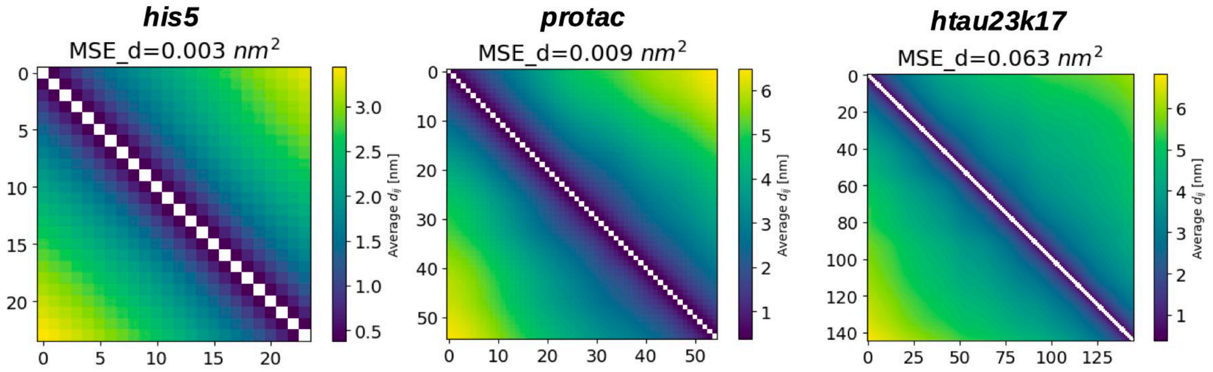

Supplementary Fig. 7. Average Cα-Cα distance maps. The figures show the average distance maps for ensembles from idpgGAN (upper triangle of the images) and MD simulations (lower triangle) for the his5, protac and htau23k17 proteins of the IDP_test set. Each cell of a map represents a residue pair and is colored according to its average d_{ij} value, where d_{ij} is the distance between Cα atoms of residues i and j in an ensemble. The MSE_d scores of the idpGAN maps with the MD ones are shown above each figure.

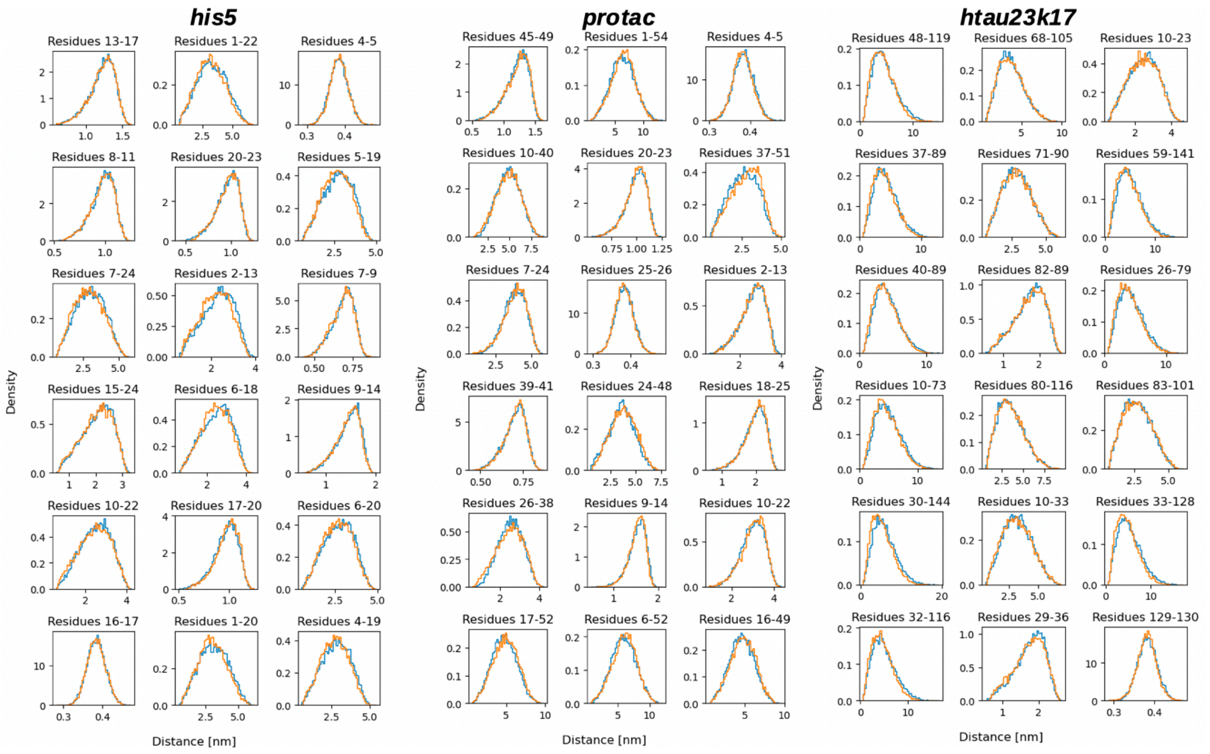

Supplementary Fig. 8. Histograms of Cα-Cα distances. The figures show distance distributions for the MD (blue color) and idpGAN (orange) ensembles for the his5, protac and htau23k17 proteins of the IDP_test set. For each protein, we show randomly selected distance distributions with the indices of the residues shown above each histogram.

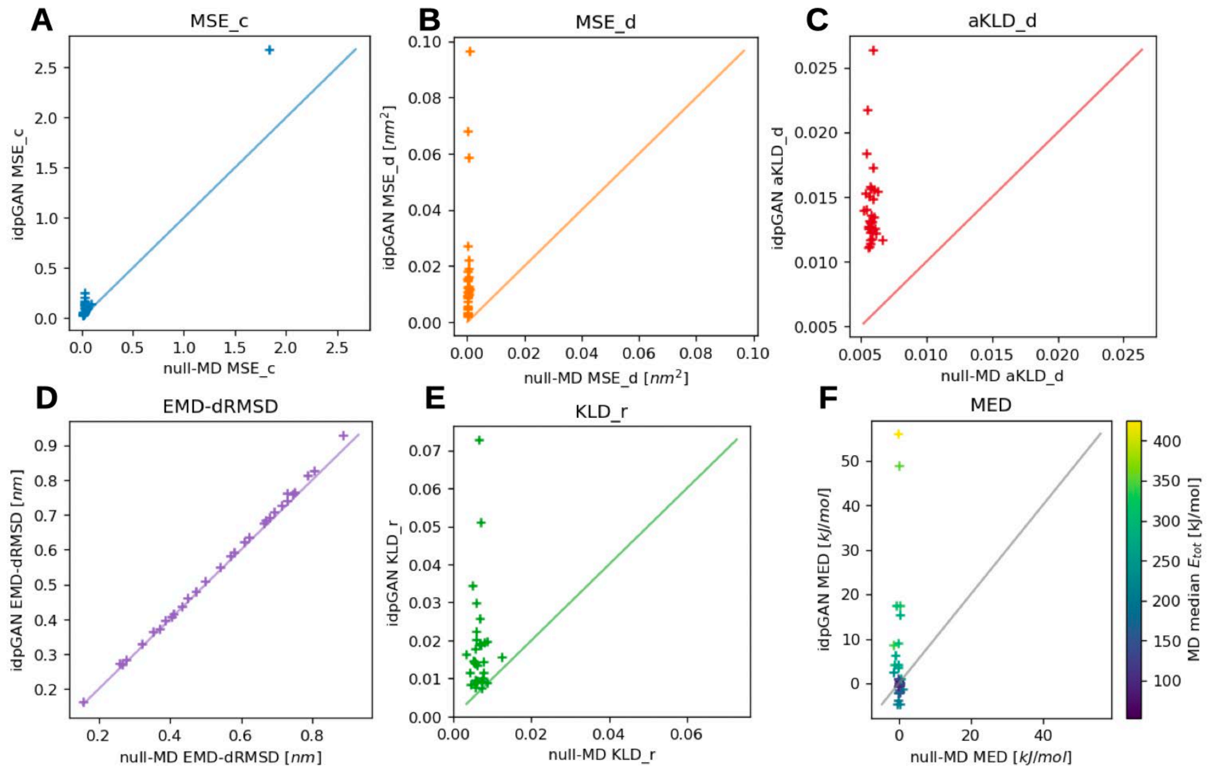

Supplementary Fig. 9. Evaluation of idpGAN and null-MD ensembles for approximating reference MD data. Results are reported for IDP_test set proteins (n = 31). **A**, **B**, **C**, **D**, and **E** show the values of MSE_c, MSE_d, aKLD_d, EMD-dRMSD and KLD_r, respectively, obtained by extracting snapshots from a long independent MD simulation (null-MD) (x-axis) and idpGAN (y-axis) for all the proteins in the set. Lower values indicate a better performance in approximating reference MD ensembles (obtained from 5 shorter MD simulations). MED values of idpGAN and null-MD ensembles are confronted in **F** with markers colored according to the median potential energy of proteins in the reference MD ensembles. Source data are provided as a Source Data file.

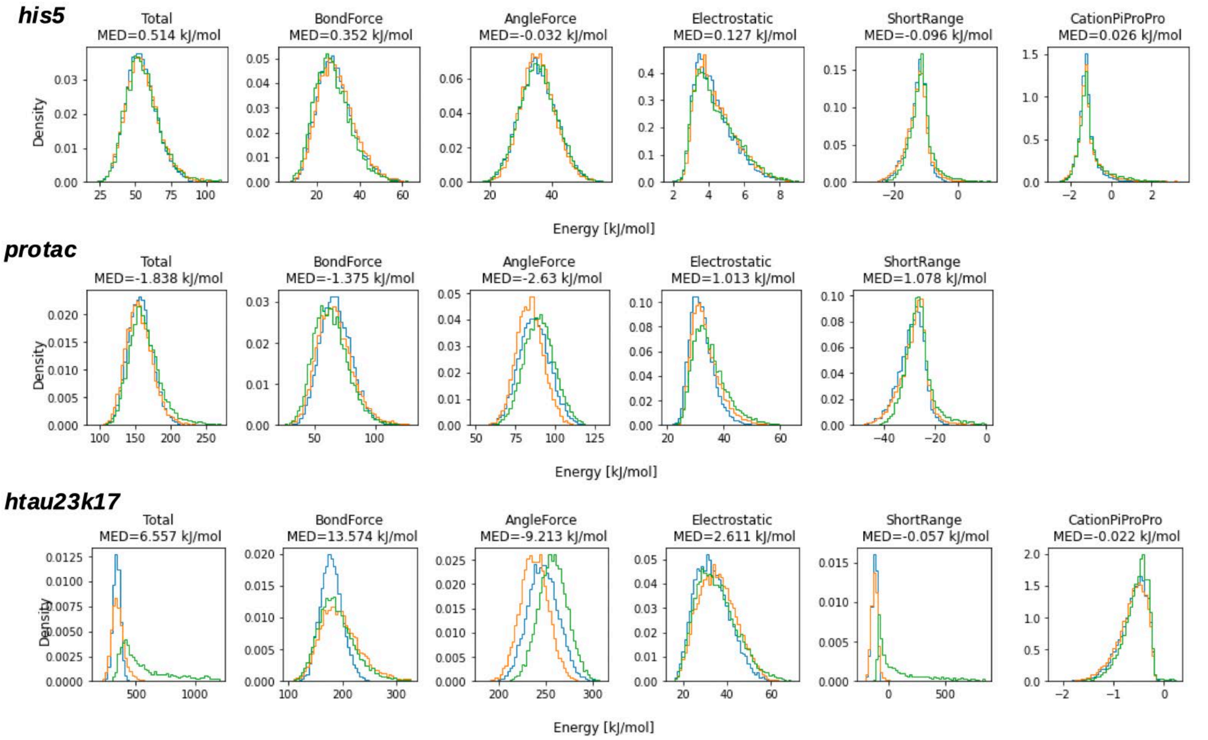

Supplementary Fig. 10. Histograms of potential energy terms for the his5, protac and htau23k17 proteins of the IDP_test. The histograms show data for the ensembles from MD (blue color), idpGAN (orange) and idpGAN trained without the additional loss term for removing steric clashes (green). The name of each energy term is shown on the top of the histograms, along with the difference in median potential energy values between the MD and idpGAN ensembles for each term. Note that for protac the cation-π term is missing, since it does not have any aromatic residues.

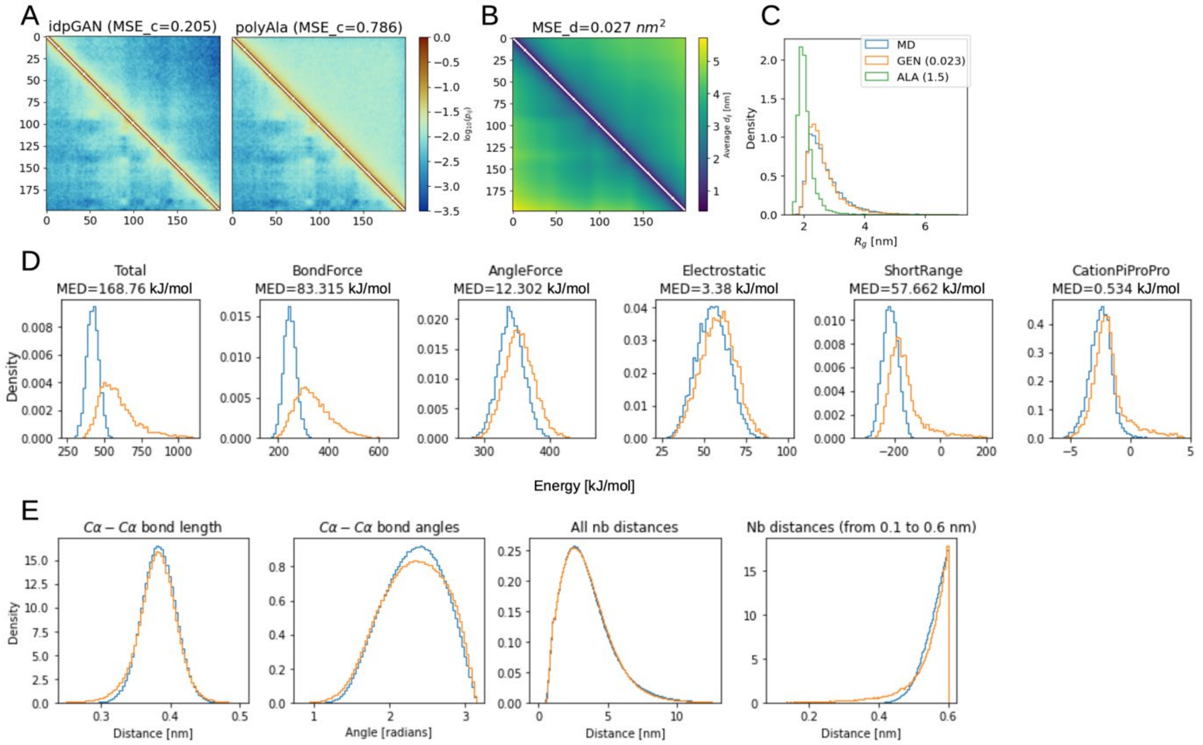

Supplementary Fig. 11. Generated ensemble of DP02478r001 (*L* = 199), an IDP of the HB_val set. **A** Contact maps of the idpGAN and polyAla ensembles (upper triangles) compared to the MD one (lower triangles), see **Fig. 2** for more details. **B** Average distance map of the MD (lower triangle) and idpGAN (upper triangle) ensembles. **C** Distributions of radius of gyration for the MD, idpGAN and polyAla ensembles. Please refer to **Fig. 2** in the main text for more information on panel **A** and **C**, and to **Supplementary Fig. 7** for panel **B**. **D** Histograms showing values of potential energy terms for the MD (blue color) and idpGAN (orange) ensembles. The difference in median potential energy between the idpGAN and MD ensembles is shown for each term. **E** Histograms showing the distribution of Cα-Cα bond lengths, bond angles and non-bonded ("nb") distances. These are the geometrical features that determine the values of the "BondForce", "AngleForce" and "ShortRange" terms respectively. The non-bonded distances histogram on the left shows all distances from 0 to 12 nm. The one on the right zooms in the 0.1 to 0.6 nm range, which contains short distances that cause high energy values for the "ShortRange" term. The overall distribution of distances is captured by idpGAN, while there are small divergences in the 0.1 to 0.6 nm range.

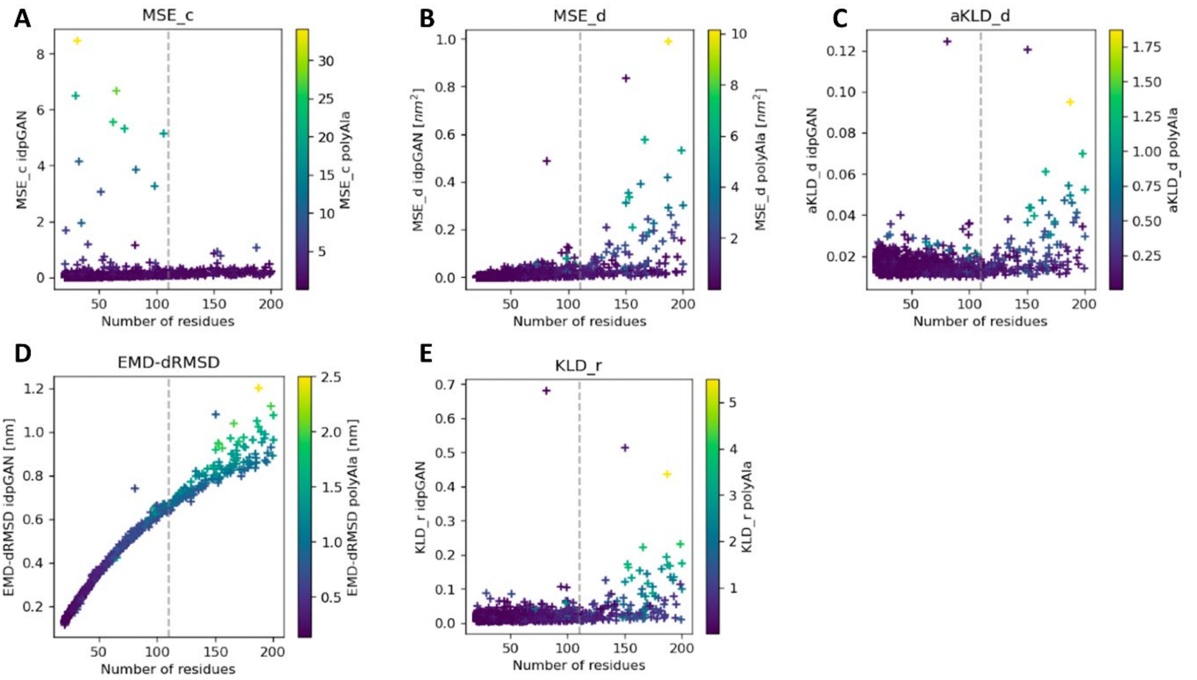

Supplementary Fig. 12. Evaluation of idpGAN ensembles for approximating MD ensembles. The panels from **A** to **E** show the values of MSE_c, MSE_d, aKLD_d, EMD-dRMSD, and KLD_r metrics respectively obtained by idpGAN for IDPs of the HB_val set as a function of protein length. The markers are colored according to the scores obtained with each metric by the polyAla approximation strategy. The dashed vertical lines represent the maximum crop length used in idpGAN training (L = 110). The scores of most evaluation metrics do not show a strong dependence on IDP length, with the exception of EMD-dRMSD. Note that this dependence arises naturally for EMD-dRMSD, since we use a fixed number of conformations n_{eval} = 10,000 to evaluate each IDP. When calculating EMD-dRMSD, each reference conformation is paired to a generated one to minimize a global dRMSD score. Since the size of the conformational space of an IDP increases with length, the expected dRMSD value between a reference conformation and the most similar one in a generated ensemble also tends to increase with IDP length if n_{eval} (the number of conformations in the generated ensemble) is kept constant. Source data are provided as a Source Data file.

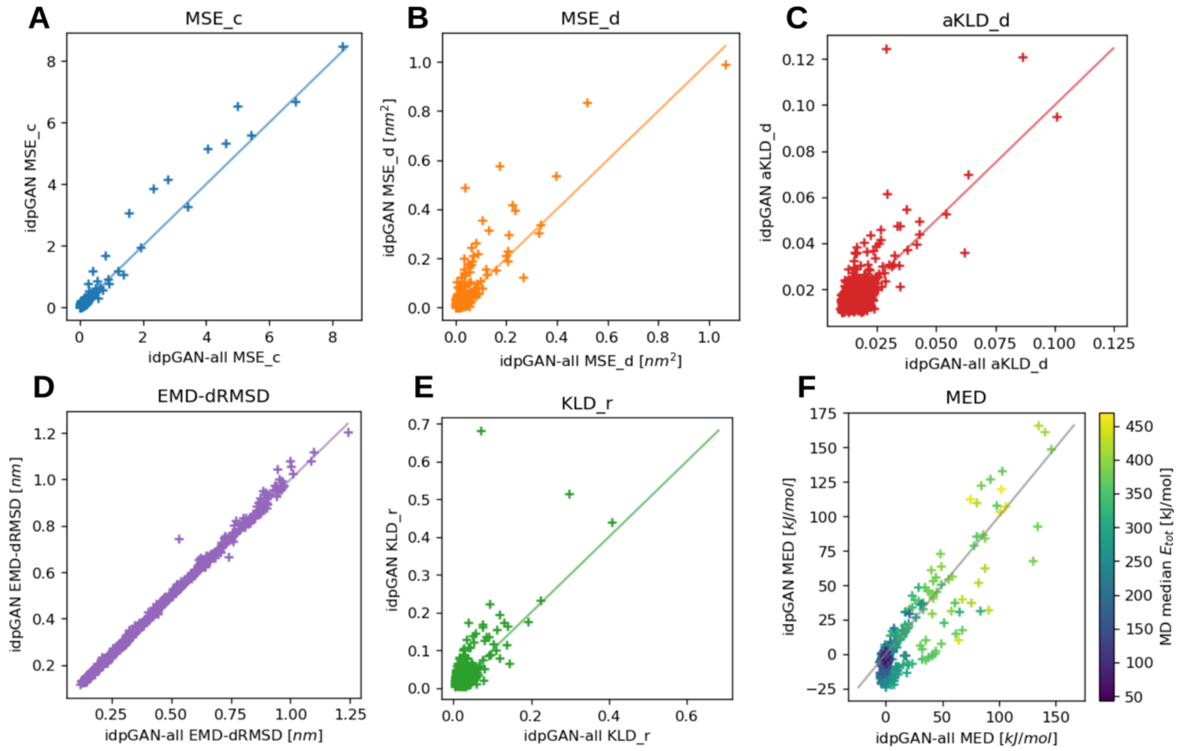

Supplementary Fig. 13. Performance of idpGAN on the proteins in its training set. Results are reported for the HB_val set proteins (n = 1,201). Each panel from **A** to **F** reports data for a different score, as in **Fig. 4**. Horizontal axes report the scores of the idpGAN-all model, which used all the HB_val proteins in its training set. Vertical axes report the scores of idpGAN models which did not use these proteins in their training sets (cf. "Methods"). Source data are provided as a Source Data file.

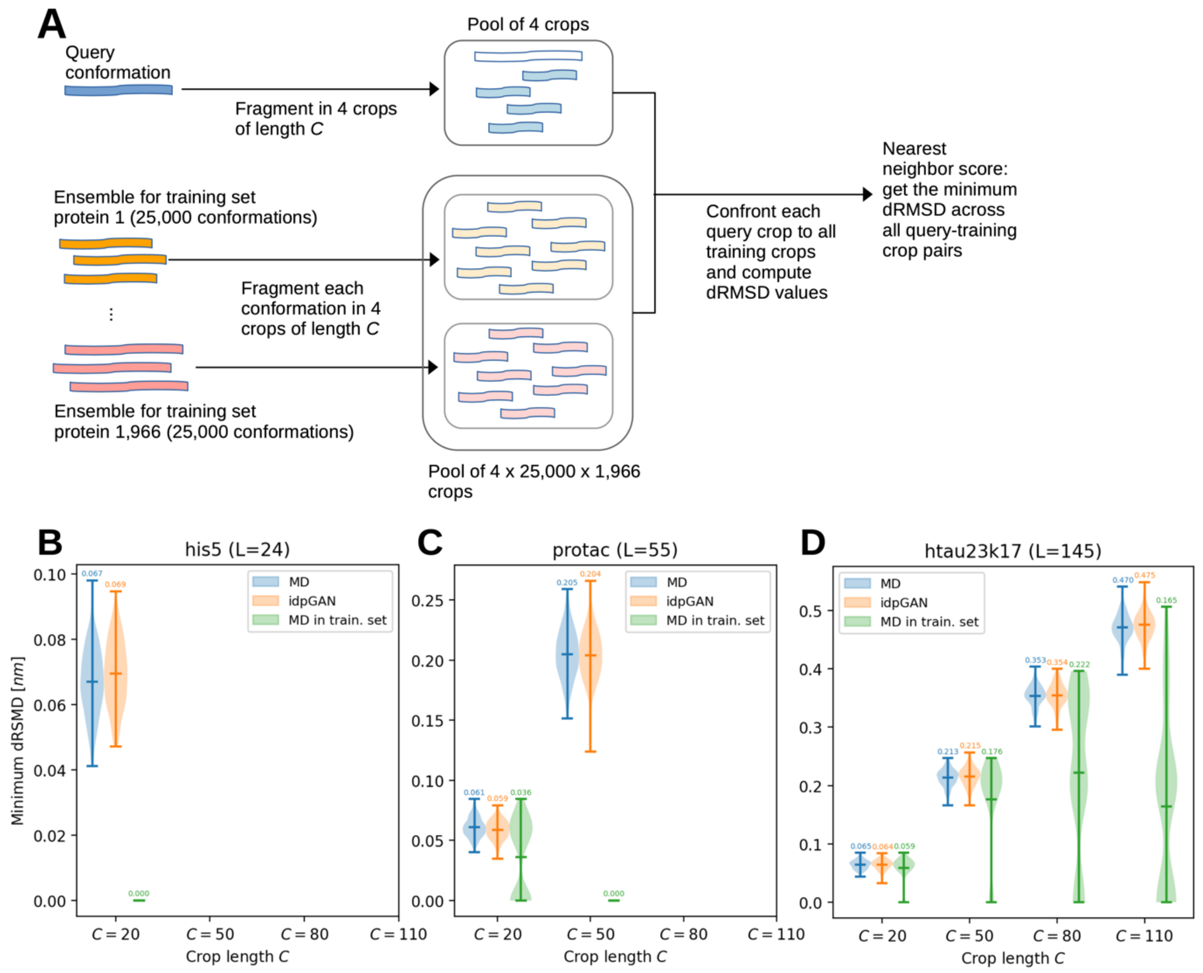

Supplementary Fig. 14. Nearest neighbor searches of idpGAN conformations over its training set. **A** illustration of the method that we use to evaluate the similarity of a CG conformation to the ones in the training set. Given a query conformation, we first randomly crop it in 4 different crops of length *C*. We also similarly process all conformations from all 1,966 proteins in the training set. We then compare via dRMSD the query crops to the entire pool of training set crops. The similarity score that we assign to the query conformation is the minimum dRMSD value obtained in all these confrontations. **B** to **D** results for the his5, protac and htau23k17 proteins of the IDP_test set. For each protein, we generated n = 200 conformations and scored their training set similarity by using crop lengths of 20, 50, 80, and 110. Data is presented as violin plots (blue color), where the center line is the mean of n = 200 scores, and extreme lines are the minimum and maximum scores respectively. Mean scores are reported above the corresponding violin plots. As a form of control, we also scored n = 200 MD conformations randomly extracted from the MD data of the three IDP_test proteins and likewise present data as violin plots (orange color). The average similarity scores of the idpGAN and MD conformations are similar for all crop lengths, indicating that idpGAN does not memorize training data. If we include the MD data of the IDP_test proteins in the training set and repeat the scoring of the MD conformations (green violin plots), the values are much lower (see the “MD in train. set” bars) because copies of the MD query conformations are now in the training set. This would be the level of similarity expected for idpGAN if it tended to directly copy training data.

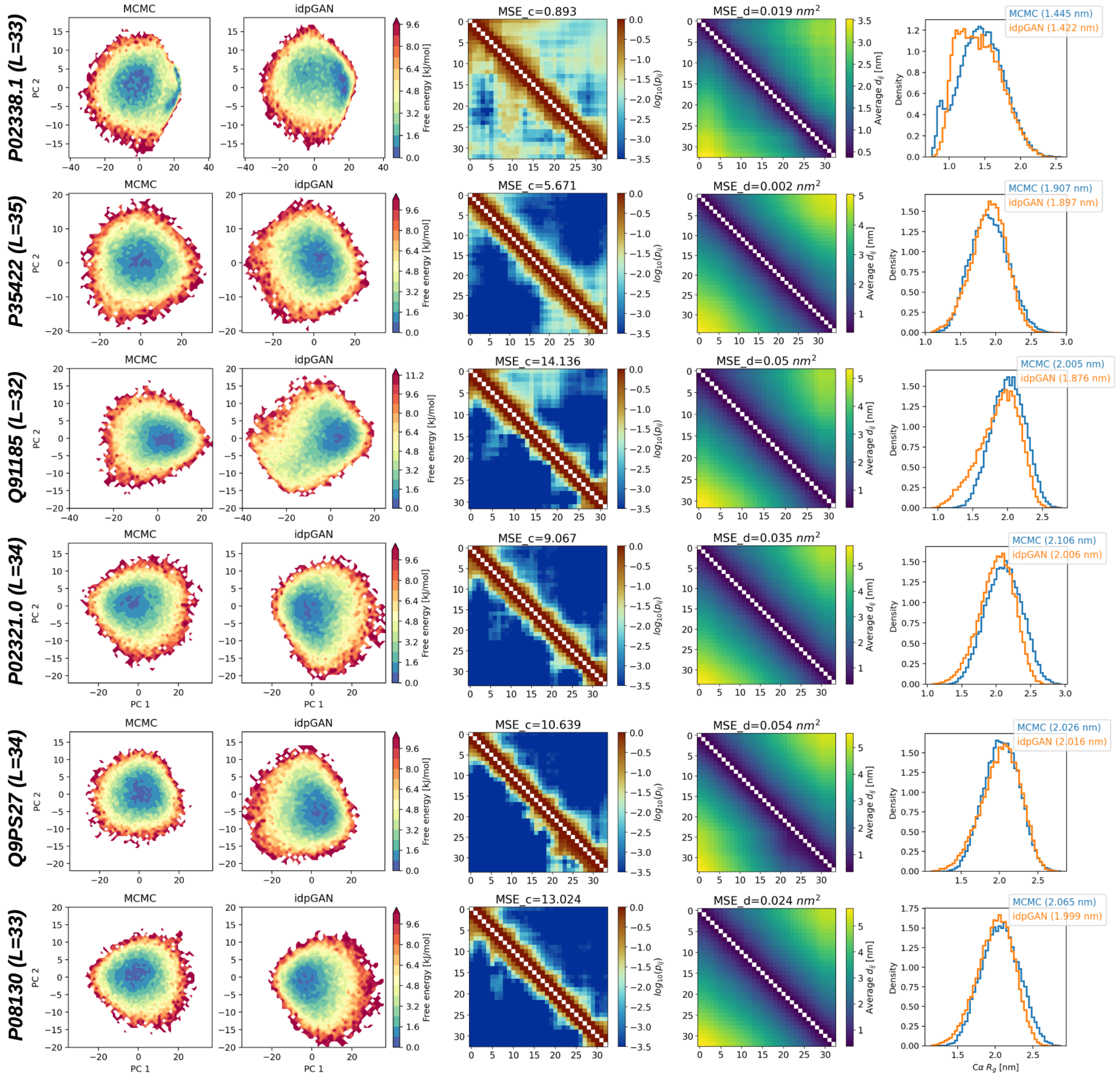

Supplementary Fig. 15. Evaluation of idpGAN on ABS_test peptides. Potential of mean force profiles, contact maps, average Cα distance maps and Cα radius-of-gyration distributions, from reference MCMC ensembles and idpGAN-generated ensembles for P02338.1, P35422, Q91185, P02321.0, Q9PS27 and P08130. See **Fig. 6** for more details.

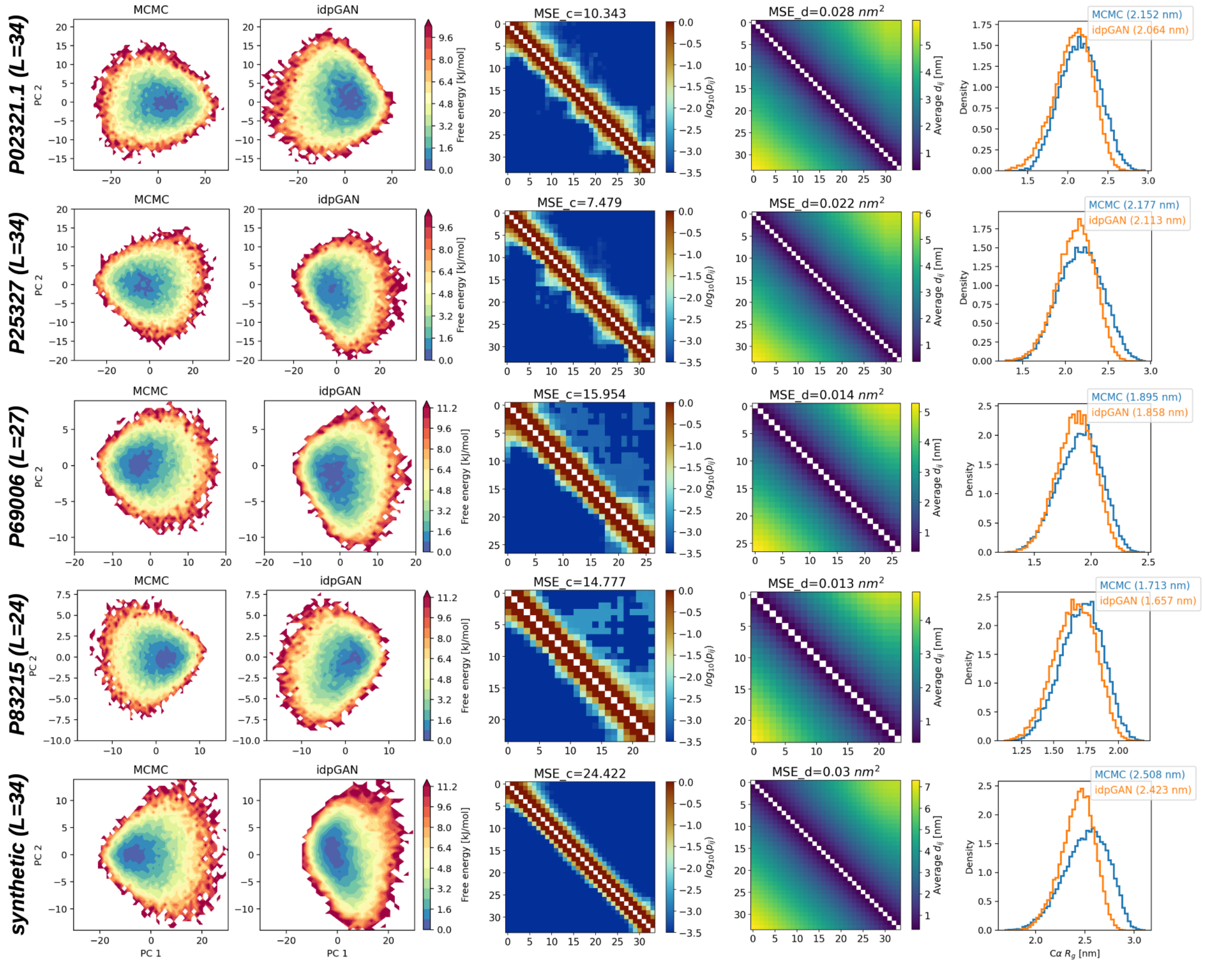

Supplementary Fig. 16. Evaluation of idpGAN on ABS_test peptides. Potential of mean force profiles, contact maps, average Cα distance maps and Cα radius-of-gyration distributions, from reference MCMC ensembles and idpGAN-generated ensembles for P02321.1, P25327, P69006, P83215 and synthetic. See **Fig. 6** for more details.

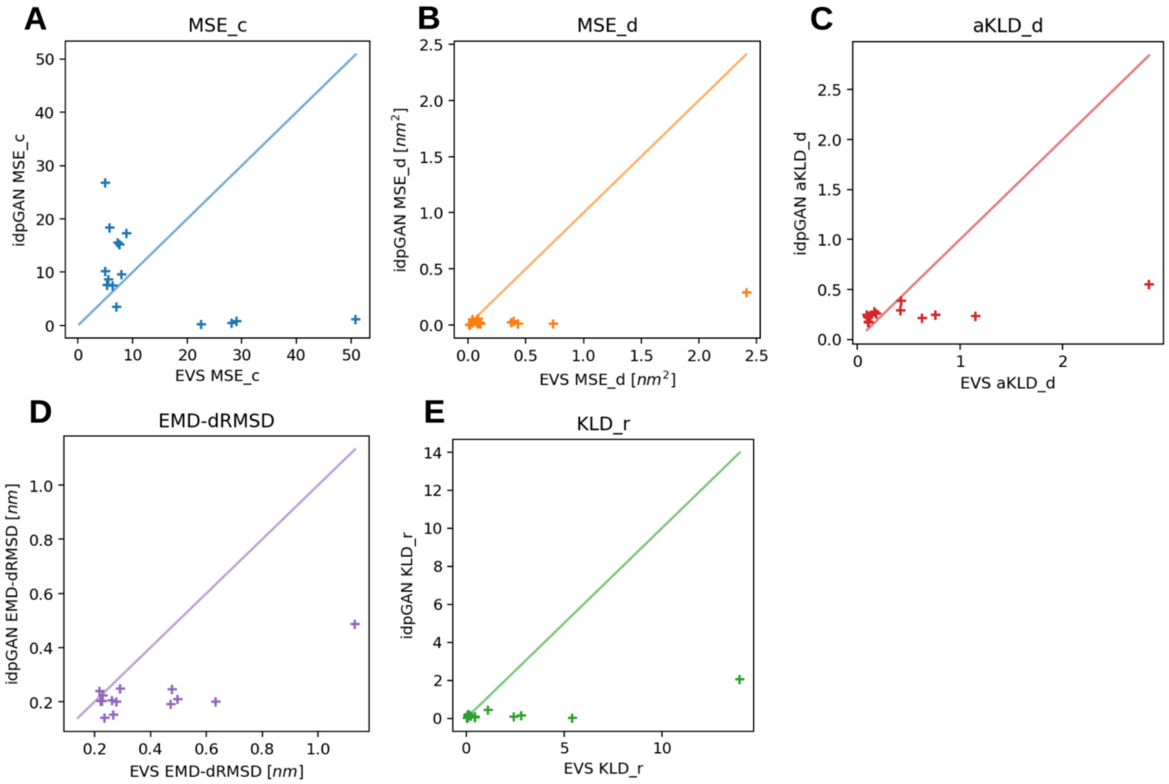

Supplementary Fig. 17. Evaluation of idpGAN and excluded solvent simulations (EVS) ensembles for approximating MCMC ABSINTH ensembles. Results are reported for ABS_test set proteins (n = 15). Each panel from **A** to **E** reports data for a different score, as in **Fig. 4**. The scores were obtained by using ensembles of 10,000 randomly sampled conformations. Source data are provided as a Source Data file.

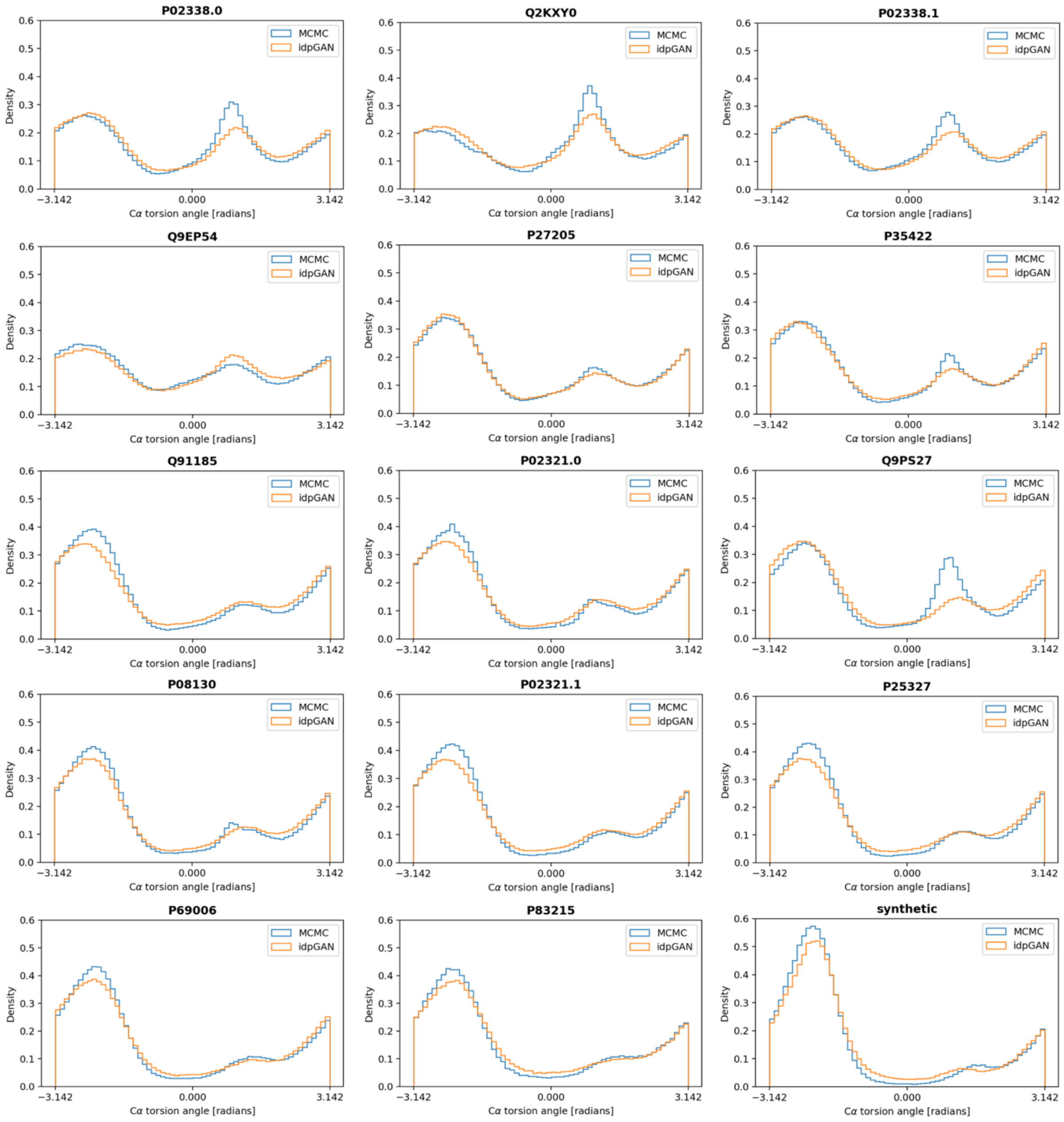

Supplementary Fig. 18. Distribution of torsion angles among four consecutive Cα atoms in the conformational ensembles of the ABS_test peptides. The plots show the distributions in the reference MCMC simulations (blue color) and in the generated idpGAN ensembles (orange color) for each peptide in the ABS_test set.

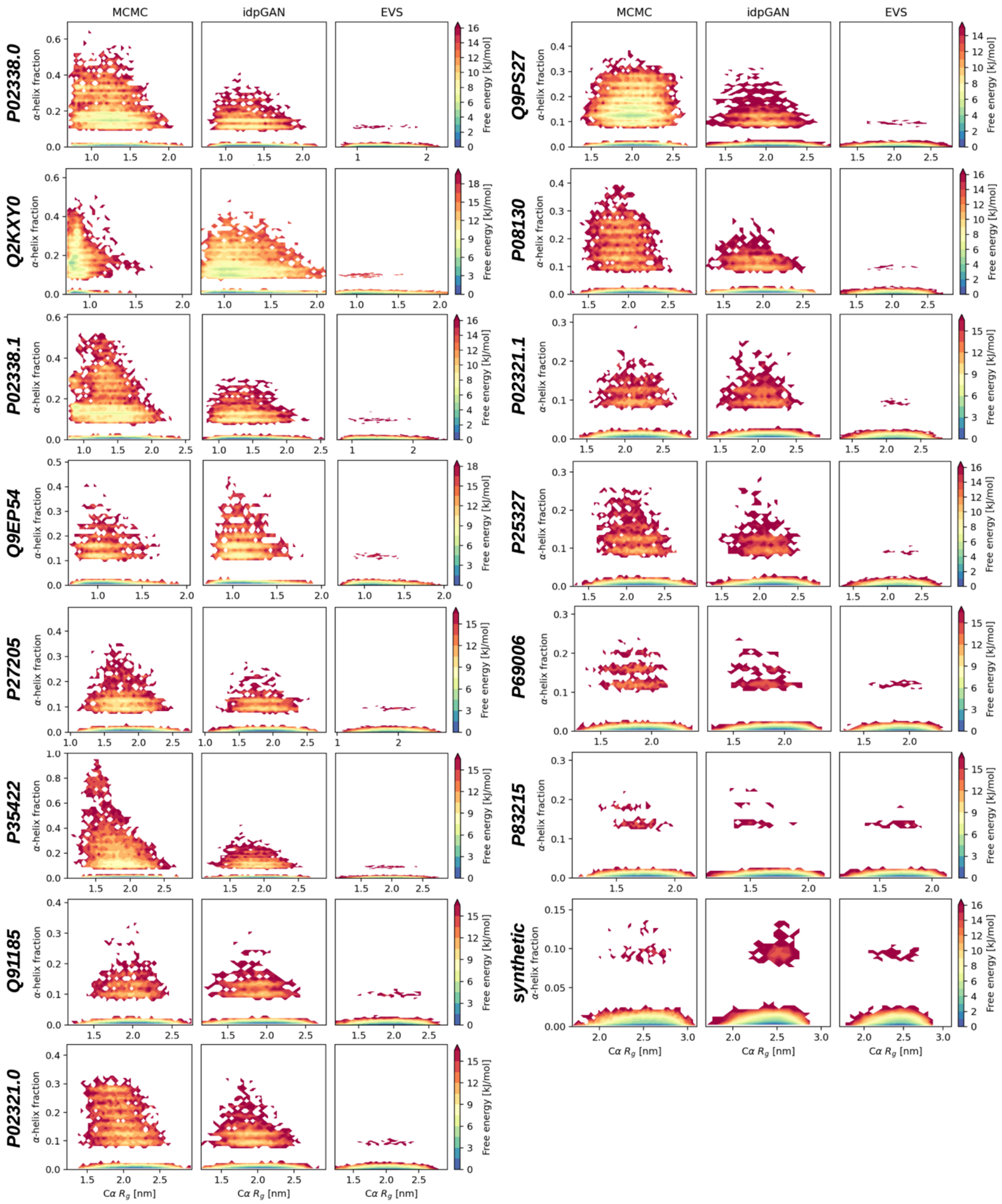

Supplementary Fig. 19. Fraction of helicity in the conformational ensembles of ABS_test peptides. The Cα radius-of-gyration and fraction of α-helical residues are used as order parameters to construct potential of mean force profiles. For each peptide, three profiles are shown: a profile for the reference MCMC ensemble (left), a profile for the idpGAN ensemble (center) and a profile for the excluded volume simulations (EVS) of the same peptide (right), which is used as a negative control. The fraction of residues in α-helix state was computed using DSSP on heavy atoms structures constructed via the MMTSB tool set from the Cα traces of the ensembles.

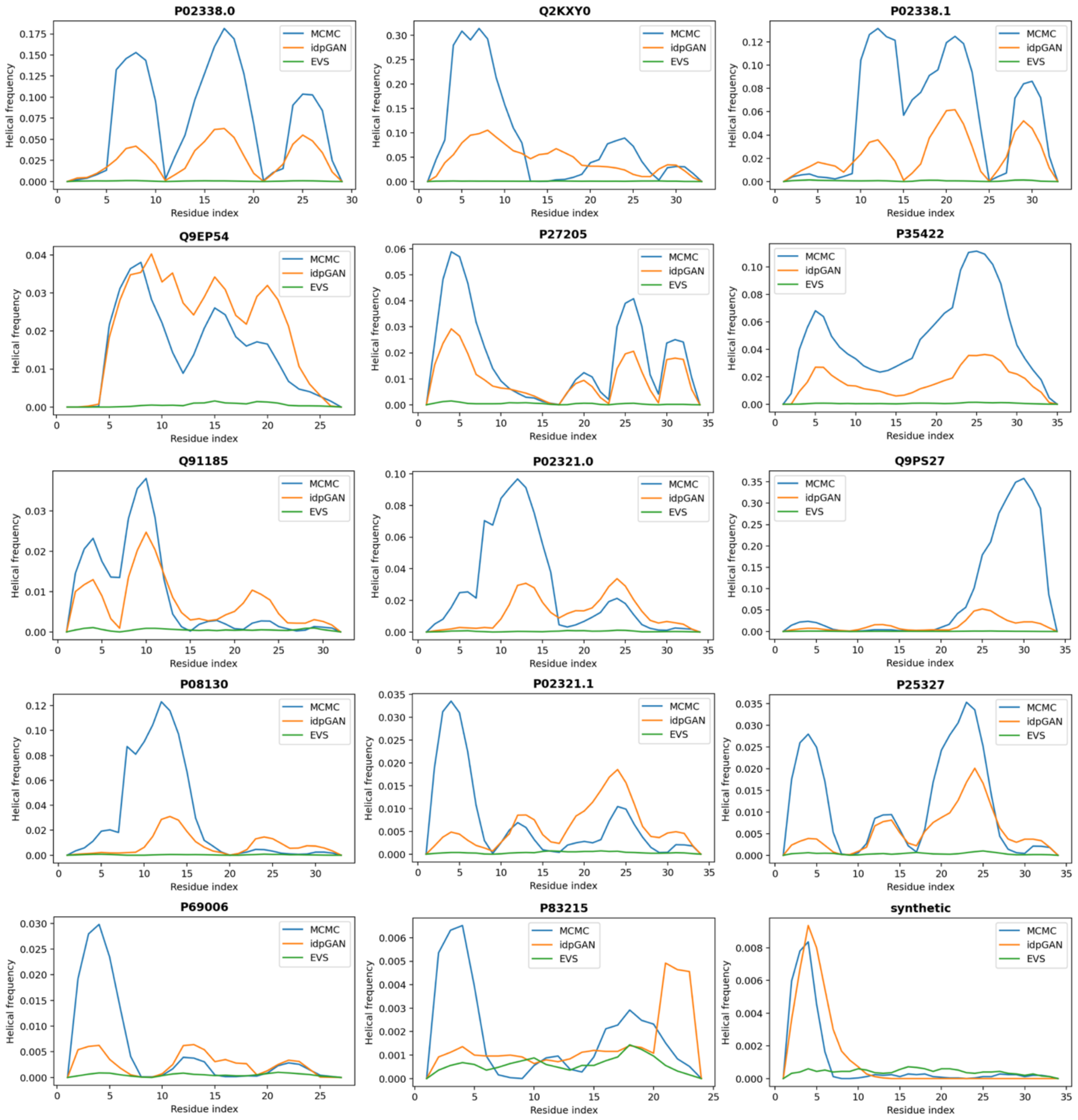

Supplementary Fig. 20. Residue-level helicity profiles in the conformational ensembles of ABS_test peptides. For each peptide of the set the figure shows the frequencies of finding a residue in α-helical state in the reference MCMC (blue), idpGAN (orange) and excluded volume simulations (EVS) ensembles (green), which are used as negative controls. The frequencies were obtained by running DSSP on heavy atoms structures constructed via the MMTSB tool set from the Cα traces of the ensembles. Source data are provided as a Source Data file.

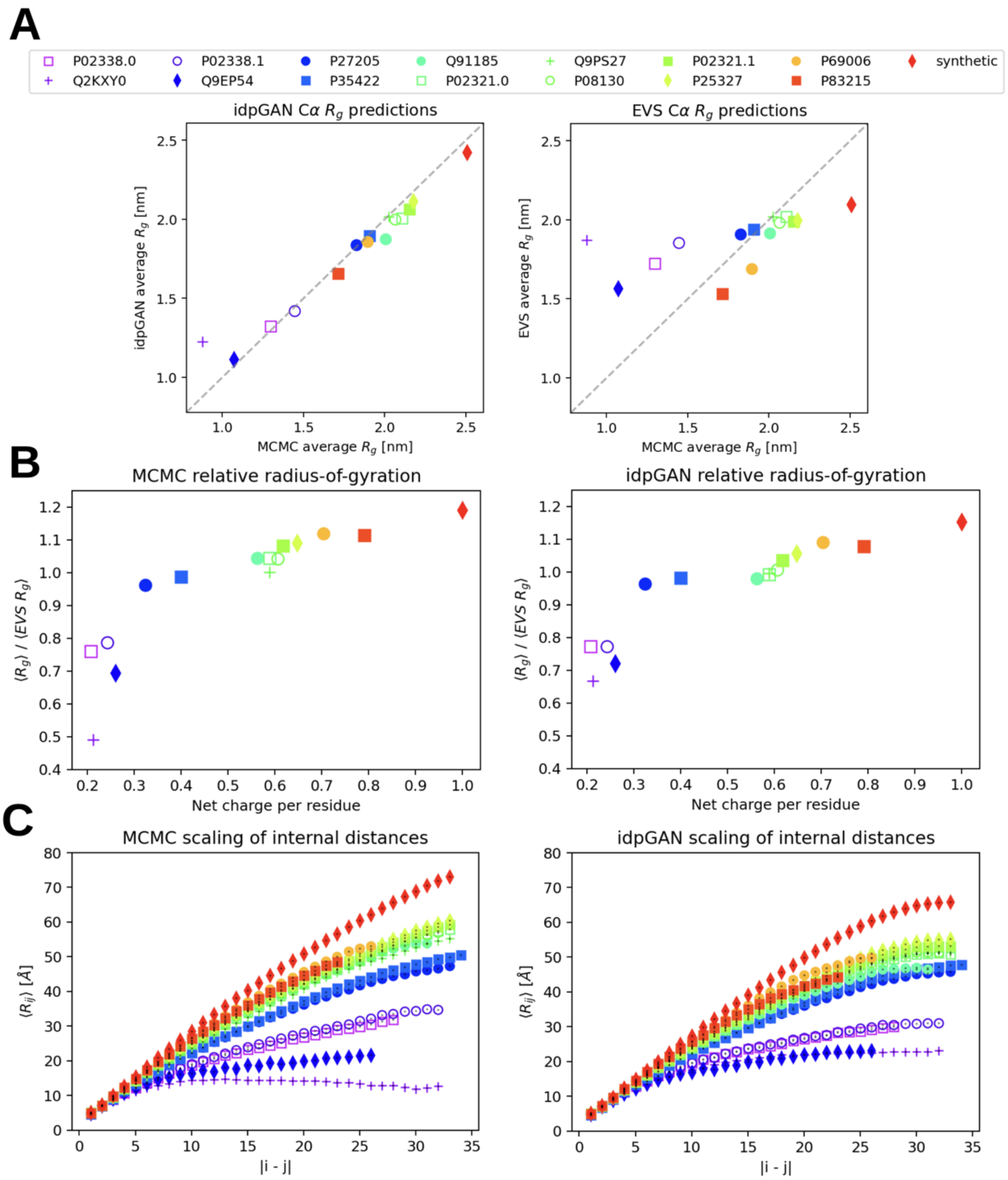

Supplementary Fig. 21. Evaluation of idpGAN for approximating ensemble properties of MCMC simulations of ABS_test peptides. **A** performance of idpGAN (left panel) and excluded solvent simulations (EVS, right panel) for approximating the average radius-of-gyration (R_g) of Cα traces of ABS_test peptides in reference MCMC simulations. **B** relative average R_g for the ABS_test peptides. The values are the ratio between the average R_g in a reference MCMC (left) or generated (right) ensemble over the average R_g in the excluded volume simulations of the same peptides. **C** average interatomic distances as a function of sequence separation in reference MCMC (right) and idpGAN (left) ensembles. Please refer to Mao et al.[1] for a formal definition of ‹ R_{ij} ›. In **A** to **C** all data was obtained by using ensembles of 25,000 randomly sampled conformations. In **B** and **C**, data for all ensembles was obtained by using all-heavy atoms structures reconstructed with the MMTSB tool set from Cα traces. These plots, which were obtained with our data, attempt to recapitulate the ones in Figure 2 and 4 of Mao et al.[1] and we used the same colors and plot styles for confrontation. Results from our MCMC simulations agree for the most part with the original ones. Source data are provided as a Source Data file.

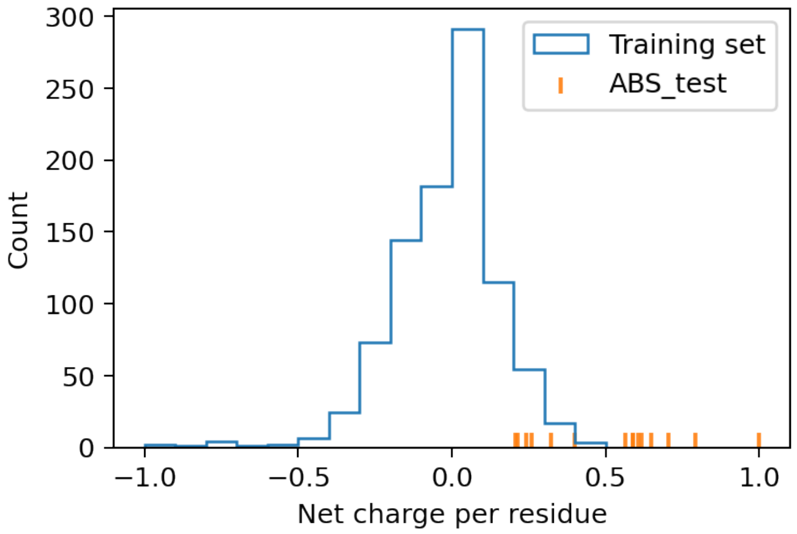

Supplementary Fig. 22. Net charge per residue in the idpGAN training set and in the ABS_test set. The figure shows a histogram of the net charge per residue values in the training set sequences used for training idpGAN on ABSINTH simulations. The orange vertical ticks are the single net charge per residue values of the 15 ABS_test peptides.

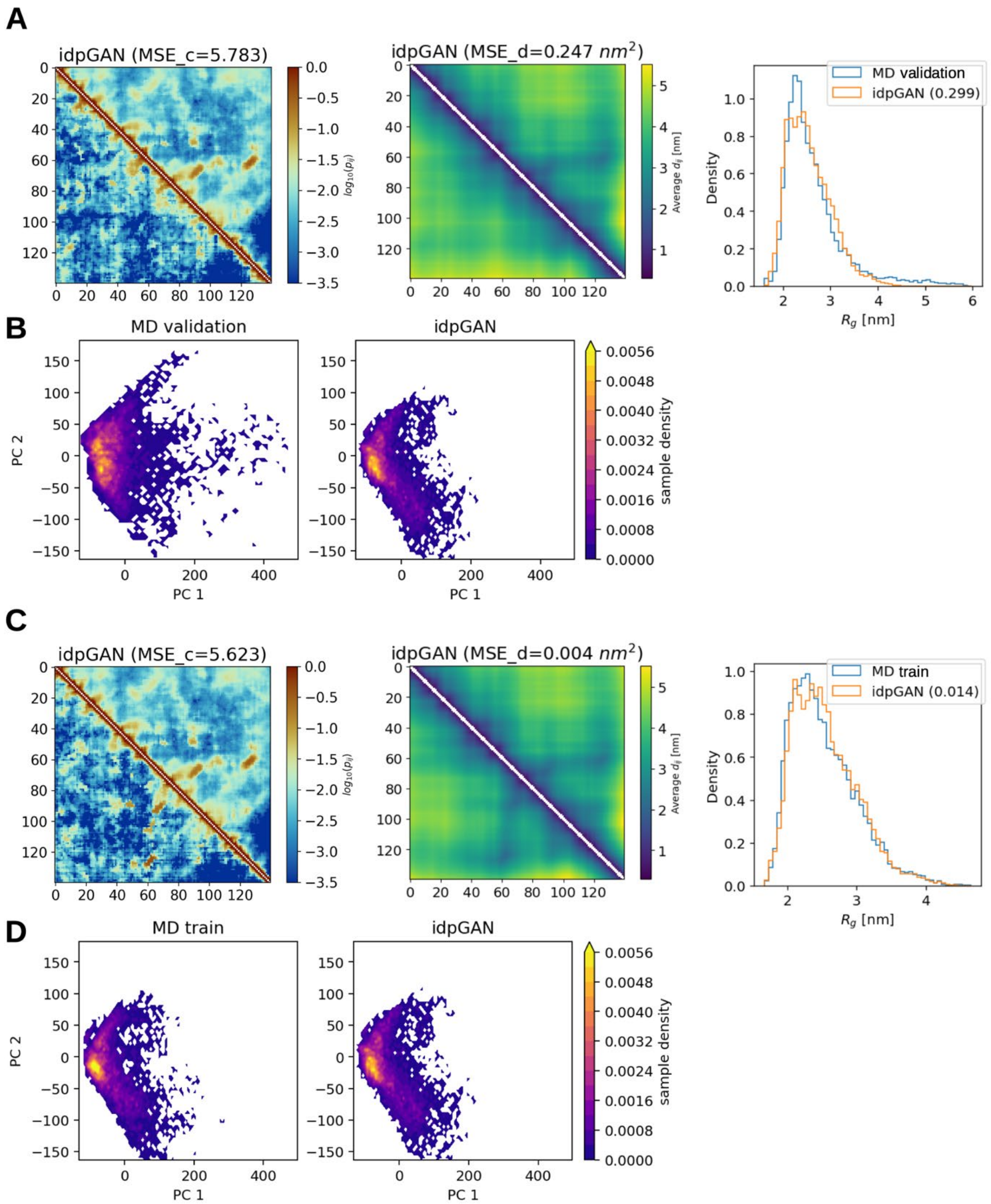

Supplementary Fig. 23. Modeling of the conformational ensemble of α-synuclein from all-atom simulations. **A**: the validation MD ensemble is confronted with the idpGAN one. Contact maps (left), average Cα distance maps (center) and Cα radius-of-gyration distributions (right) are shown. The idpGAN contact map (upper triangle) is confronted with the MD map (lower triangle), see **Fig. 2** for details. The idpGAN average Cα distance map (upper triangle) is similarly confronted with the corresponding MD map (lower triangle). The Cα radius-of-gyration distributions from MD and idpGAN are compared (their KLD_r value is shown in brackets). **B**: distribution of validation MD and idpGAN ensembles in PCA space. A PCA model was fit on the validation MD data by featurizing conformations as the set of all their Cα interatomic distances. Validation MD and idpGAN conformations were then projected onto the first two principal components. **C** and **D**: the training MD ensemble is confronted with the idpGAN one. The panels are organized in the same way of panels **A** and **B**, respectively. In **D**, the training MD and idpGAN conformations were projected onto the first two principal components used in **B**.

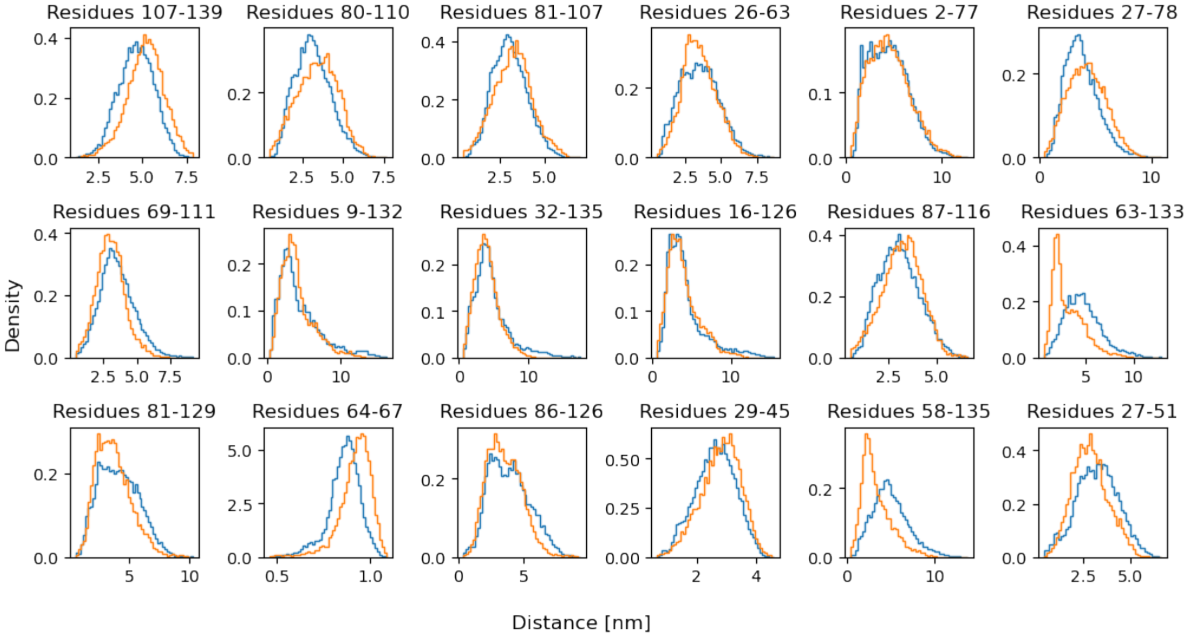

Supplementary Fig. 24. Histograms for Cα-Cα distances for all-atom α-synuclein conformations. Data for the validation MD (blue color) and idpGAN (orange) ensembles is reported. 18 distance distributions between randomly selected residues are shown (the indices of the residues are indicated above each histogram).

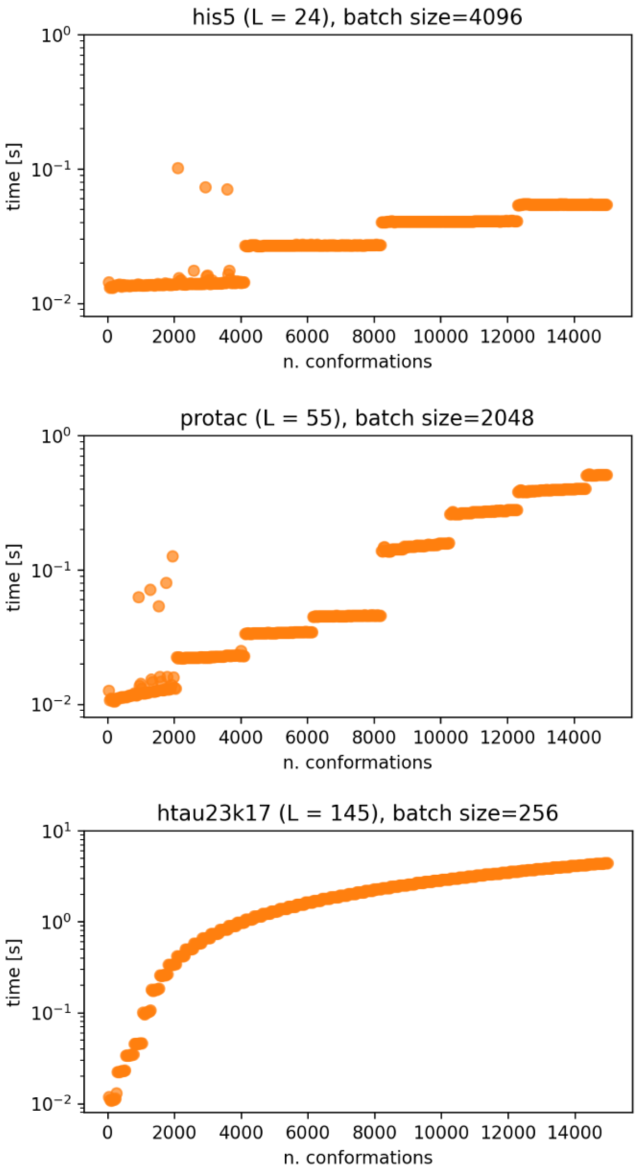

Supplementary Fig. 25. GPU time used by the G network to generate different numbers of conformations. We show data for the his5, protac and htau23k17 proteins of the IDP_test set. Beside the name of the protein, its number of amino acids L and the batch size used to generate all the samples in the ensembles are shown. Please refer to the "Methods" section in the main text and **Supplementary Table 6** for details. Source data are provided as a Source Data file.

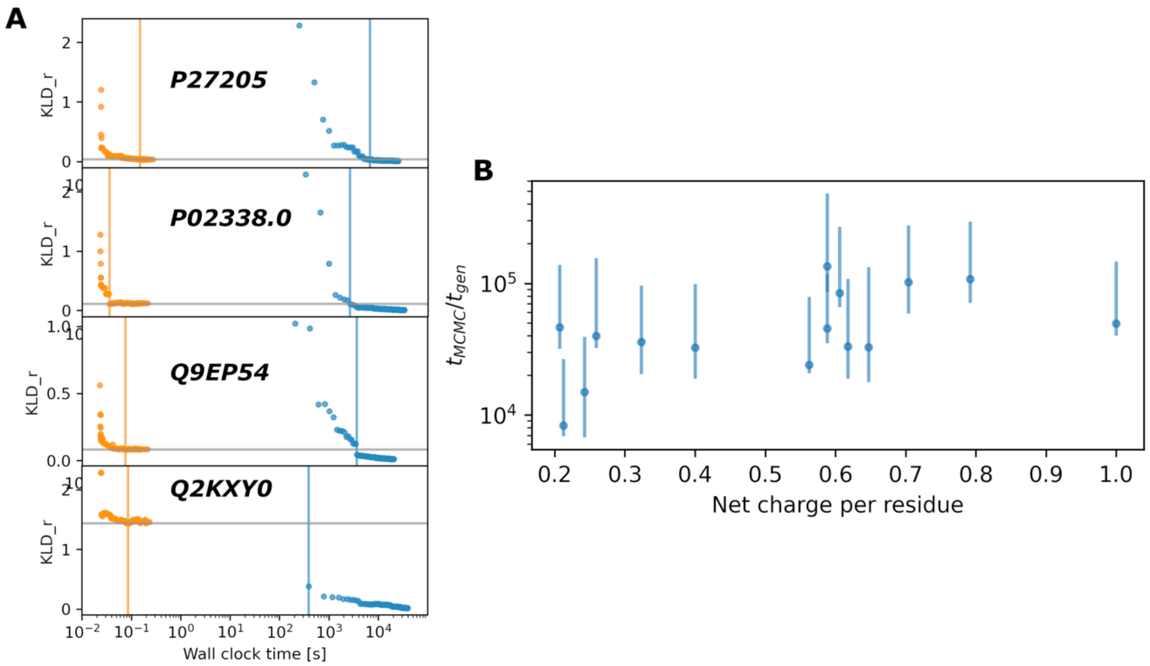

Supplementary Fig. 26. Evaluation of idpGAN sampling efficiency compared to ABSINTH MCMC simulations of ABS_test peptides. A KLD_r between idpGAN and reference MCMC ensembles (orange data points) and between MCMC ensembles from parallel simulations and reference MCMC ensembles (blue) as a function of wall clock time. The reference ensemble of a peptide, called E_{MCMC}, is constituted by 25,000 randomly-extracted conformations from 20 independent MCMC simulations. To measure the sampling efficiency of MCMC simulations, we consider ensembles $E_{MCMC,m}$ that contain m conformations. These conformations are selected from the beginning of the 20 independent MCMC simulations (e.g.: a $E_{MCMC,60}$ ensemble contains the first 3 snapshots from each of the 20 simulations of a peptide). Orange vertical lines indicate the GPU wall clock time (t_{gen}) at which the generator reaches a plateau KLD_r_{top} value, which is marked by a gray horizontal line. In this experiment, the GPU time corresponds to the time needed to run sequentially the idpGAN generator and the mirror image selector network. The blue vertical lines indicate the total CPU wall clock time (t_{MCMC}) employed by the 20 parallel simulations to sample enough conformations to surpass the KLD_r_{top} value. Data is shown for the P27205, P02338.0, Q9EP54 and Q2KXY0 peptides with t_{gen} values of 1.5×10^{-1} s, 3.6×10^{-2} s, 7.6×10^{-2} s, and 8.6×10^{-2} s and t_{MCMC} values of 6.7×10^{3} s, 2.6×10^{3} s, 3.7×10^{3} s, and 3.9×10^{2} s respectively. **B** t_{MCMC}/t_{gen} ratios for all peptides of the ABS_test set are plotted as a function of net charge per residue. Data are presented as mean values of 10 runs (blue data points) and the bars report minimum and maximum values across the runs. In **A** and **B** for all experiments with idpGAN we used a batch size of 4096. Source data are provided as a Source Data file.

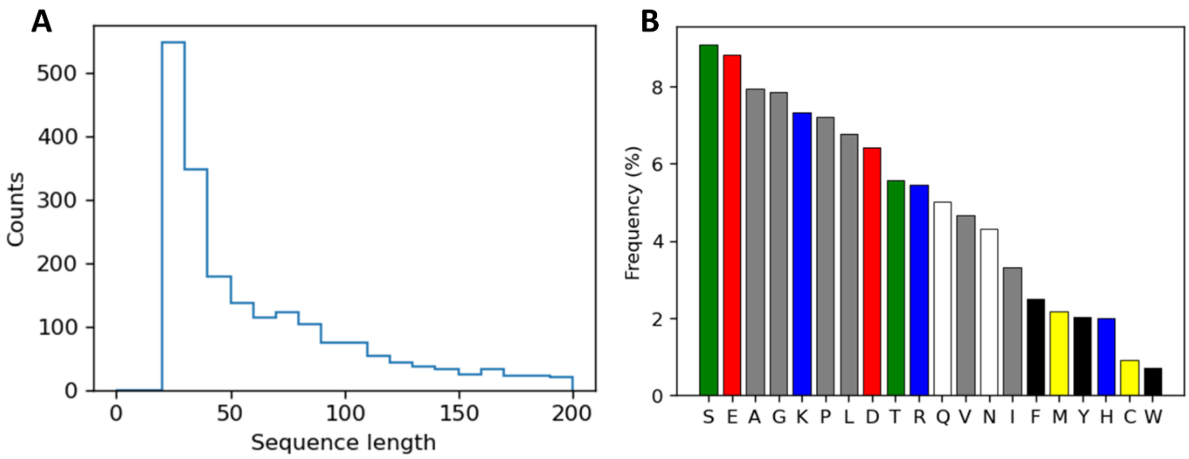

Supplementary Fig. 27. Properties of the 1,966 intrinsically disordered regions (IDRs) sequences from DisProt in the training set of idpGAN. A: Histogram of the sequence lengths of the IDRs. **B:** Amino acid frequencies in the IDRs. The amino acids are colored according to their physiochemical characteristics: green is for "small hydroxy", red is for "acidic", gray is for "aliphatic", blue is for "basic", white is for "amide", black is for "aromatic", yellow is for "sulfur". Colors were adopted from a similar plot in https://www.ebi.ac.uk/uniprot/TrEMBLstats showing amino acid frequencies in the whole TrEMBL database.

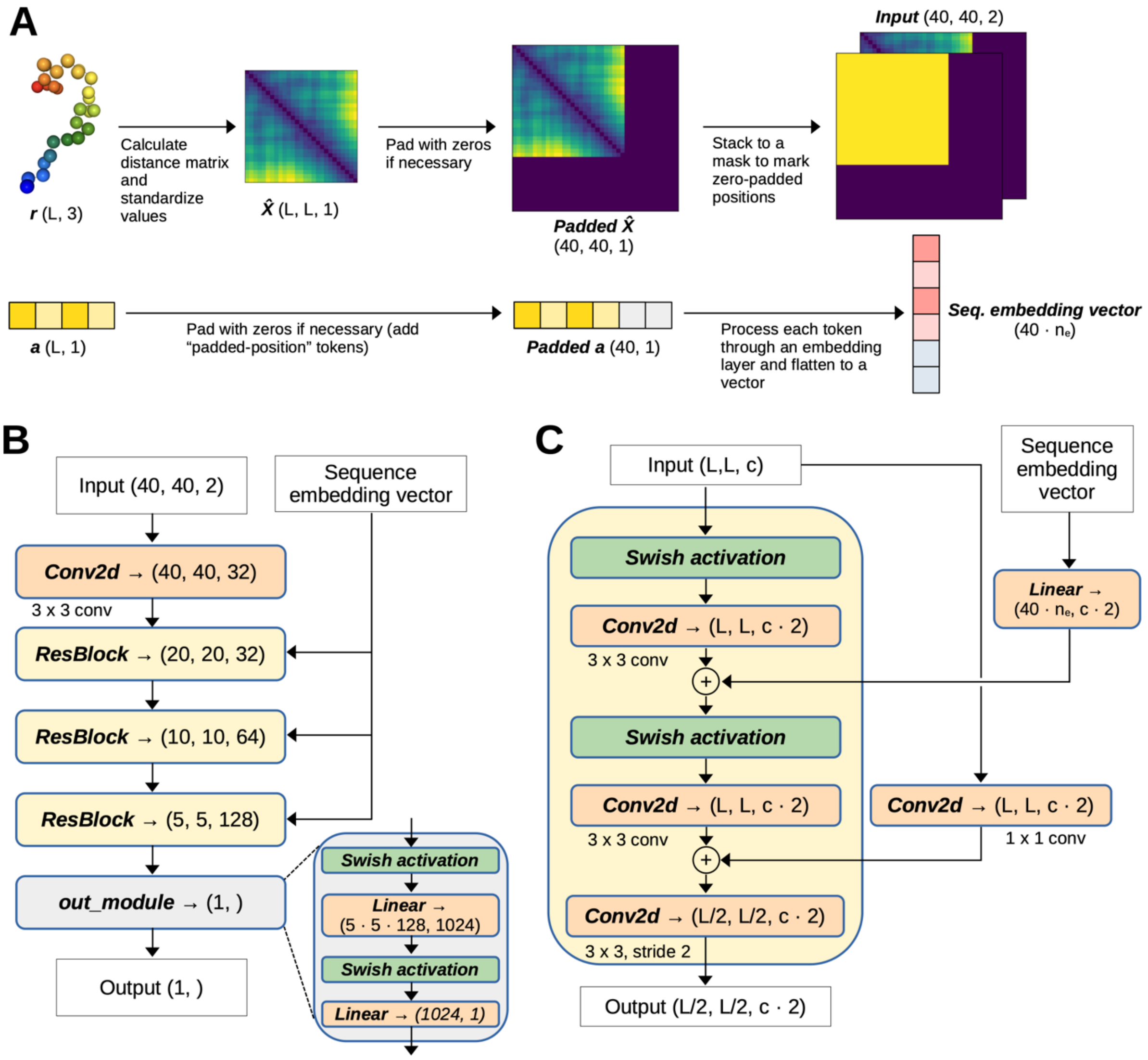

Supplementary Fig. 28. Details of the discriminator network to train idpGAN on ABSINTH simulations. A: Preparation of the input for the D network. The Cα distance matrix of a peptide is processed using a non-trainable standardization layer as for the D networks used in CG modeling (see "Methods" in the main text). If the peptide has less than 40 residues, the distance matrix is padded with zeros to obtain a 40 × 40 matrix. This matrix is then stacked to a 40 × 40 mask in which positions originated from zero-padding contain 0, and the remaining positions contain 1. The resulting tensor has shape 40 × 40 × 2 and is part of the input. The network also receives as input an amino acid sequence embedding vector of dimensions 40 · n_e (with $n_e = 16$). This vector is constructed by first processing the amino acid sequence of the peptide (which may be padded with "padded-position" tokens to reach a length of 40) through an embedding layer with vocabulary size of 21 (20 amino acids and a "padded-position" token) and with output size of n_e. The embeddings of all position are then concatenated to obtain the sequence embedding vector. **B**: architecture of the D network. The network has a 2d convolutional network architecture with 3 residual blocks. The 40 × 40 × 2 input is processed by the residual blocks and a fully-connected module to produce a scalar output. The sequence embedding vector is injected at each residual block. **C**: details of the residual blocks. The sequence embedding vector is linearly projected to the same dimension of the hidden feature map and added to each position of it.

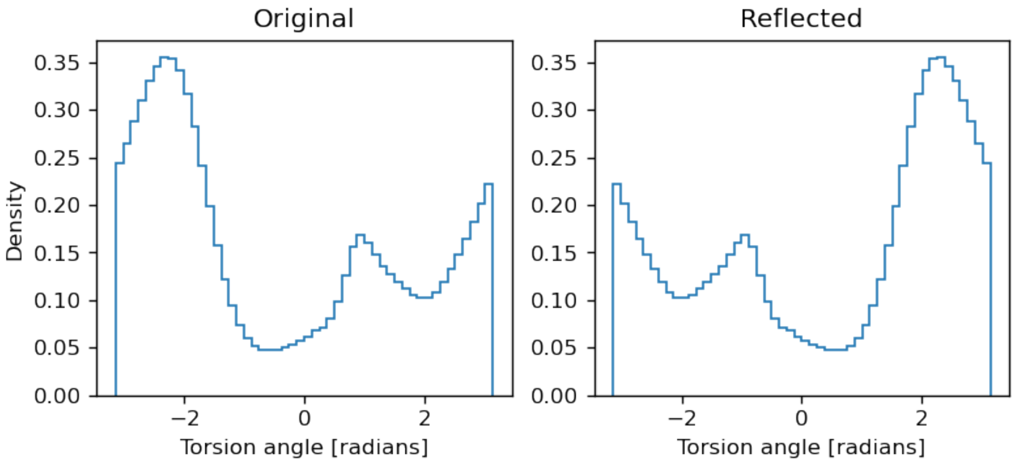

Supplementary Fig. 29. Distribution of torsion angles among consecutive Cα atoms in ABSINTH simulations. The distribution is from the conformations of all peptides from the ABS_test set. On the left panel we show data from the original conformations, on the right panel data from the mirror images of the original conformations.

Supplementary Table 1. Sequences of the proteins in the IDP_test set.

Name	Len.	R_g (nm)	Sequence	Ref.
his5	24	1.38	DSHAKRHHGYKRKFHEKHHSHRGY	[2]
ak37	37	1.69	AAKAAAAKAAAAKAAAAKAAAAKAAAAKAAAAKAAGY	[3]
n49	38	1.37	GCQTSRGLFGNNNTNNINNSSSGMNNASAGLFGSKPFA	[4]
cytc_nter	39	1.84	MIFFMVMPIMIGGFGNWLVPLMIGAPDMAFPRMNNSFWL	[3]
nls	46	1.63	ACETNKRKREQISTDNEAKMQIQEEKSPKKKRKKRSSKANKPPEFA	[4]
protac	55	3.00	CEEGGEEEEEEEEGDGEEEDGDEDEEAESATGKRAAEDDEDDDVDTKKQKTDEDC	[5]
protan	55	2.55	CDAAVDTSSEITTKDLKEKKEVVEEAENGRDAPANGNANEENGEQEADNEVDEEC	[5]
protein_g	56	2.30	MQYKLALNGKTLKGETTTEAVDAATAEKVFKQYANDNGVDGEWAYDDATKTFAVTE	[6]
drk_sh3	59	2.19	MEAIAKHDFSATADDELSFRKTQILKILNMEDDSNWYRAELDGKEGLIPSNYIEMKNHD	[7]
in	60	2.16	GSHCFLDGIDKAQEEHEKYHSNWRAMASDFNLPPVVAKEIVASCDKCQLKGEAMHGQVDC	[8]
protein_l	64	1.65	MEEVTIKANLIFANGSTQTAEFKGTFEKATSEAYAYADTLKKDNGEWTVDVADKGYTLNIKFAG	[9]
actr	71	2.51	GTQNRPLLRNSLDDLVGPPSNLEGQSDERALLDQLHTLLSNTDATGLEEIDRALGIPELVNQGQALEPKQD	[10]
csp_tm	67	1.47	GPGMCRGKVKFFDSKKGYGFITKDEGGDVFVHFSAIEMEGFKTLKEGQVVEFEIQEGKKGGQAAHVKVVEC	[5]
ubiquitin	76	2.52	MQIFVKTLTGKTITLEVEPSDTIENVKAKIQDKEGIPPDQQRLIWAGKQLEDGRTLSDYNIQKESTLHLVLRLRGG	[3]
sh4	85	2.82	MGSNKSKPKDASQRRRSLEPAENVHGAGGGAFPASQTPSKPASADGHRGPSAAFAPAAAEPKLFGGFNSSDTVTSPQRAGPLAGG	[11]
sic1	90	3.00	MTPSTPPRSRGTRYLAQPSGNTSSSALMQGQKTPQKPSQNLVPVTPSTTKSFKNAPLLAPPNSNMGMTSPFNGLTSPQRSPFPKSSVKRT	[12]
p53	93	2.87	MEEPQSDPSVEPPLSQETFSDLWKLLPENNVLSPLPSQAMDDLMLSPDDIEQWFTEDPGPDEAPRMPEAAPPVAPAPAAPTPAAPAPAPSWPL	[13]
ibb	99	2.52	GCTNENANTPAARLHRFKNKGKDSTEMRRRRIEVNVELRKAKKDDQMLKRRNVSSFPDDATSPLQENRNNQGTVNWSVDDIVKGINSSNVENQLQATFA	[4]
ul11	104	2.43	MGLSFSGTRPCCCRNNVLITDDGEVVSLTAHDFDVVDIESEEEGNFYVPPDMRGVTRAPGRQRLRSSDPPSRHTHRRTPGGACPATQFPPPMSDSEWSHPQFEK	[14]
r15	114	2.33	KLKEACKQQNFNTGIKDFDFWLSEVEALLASEDYGKDLASVNNLLKKHQLLEADISAHEDRLKDLNSQADSLMTSSAFDTSQVKDKRETINGRFQRIKCMAAARRAKLNESHRL	[8]
nul	114	2.66	GCGFKGFDTSSSSSNSAASSSFKFGVSSSSSGPSQTLTSTGNFKFGDQGGFKIGVSSDSGSINPMSEGFKFSKPIGDFKFGVSSESKPEEVKKDSKNDNFKFGLSSGLSNPVFA	[4]
r17	118	2.37	GSRLEESCEYQQFVANVEEEEAWINEKMTLVASEDYGDTLAAIQGLLKKHEAFETDFTVHKDRVNDVAANGEDLIKKNNHHVENITAKMKGLKGKVSDLECAAAQRKAKLDENSAFLQ	[8]
erm	122	3.96	MDGFYDQQVPFMVPGKSRSEECRGRPVIDRKRKFLDTDLAHDSEELFQDLSQLQEAWLAEAQVPDDEQFVPDFQSDNLVLHAPPPTKIKRELHSPSSELSSCSHEQALGANYGEKCLYNYCA	[15]
rnasea	124	3.36	KETAAAKFERQHMDSSTSAASSSNYCNQMMKSRNLTKDRCKPVNTFVHESLADVQAVCSQKNVACKNGQTNCYQSYSTMSITDCRETGSSKYPNCAYKTTQANKHIIVACEGNPYVPVHFDASV	[16]
hnhe1cdt	131	3.63	MVPAHKLDSPTMSRARIGSDPLAYEPKEDLPVITIDPASPQSPESVDLVNEELKGKVLGLSRDPAKVAEEDEDDDGGIMMRSKETSSPGTDDVFTPAPSDSPSSQRIQRCLSDPGPHPEPGEGEPFFPKGQ	[10]
snase	136	2.12	ATSTKKLHKEPATLIKAIDGDTVKLMYKGQPMTFRLLLVDTPETKHPKKGVEKYGPEASAFTKKMVENAKKIEVEFDKGQRTDKYGRGLAYIYADGKMVNEALVRQGLAKVAYVYKPNNTHEQHLRKSEAQAKKEK	[17]
asynuclein	140	3.30	MDVFMKGLSKAKEGVVAAAEKTKQGVAEAAGKTKEGVLYVGSKTKEGVVHGVATVAEKTKEQVTNVGGAVVTGVTAVAQKTVEGAGSIAAATGFVKKDQLGKNEEGAPQEGILEDMPVDPDNEAYEMPSEEGYQDYEPEA	[18]
fhua	143	3.34	ESAWGPAATIAARQSATGTKTDTPIQKVPQSISVVTAEEMALHQPKSVKEALSYTPGVSVGTRGASNTYDHLIIRGFAAEGQSQNNYLNGLKLQGNFYNDAVIDPYMLERAEIMRGPVSVLYGKSSPGGLLNMVSKRPTTEPL	[16]
htau23k17	145	3.60	MSSPGSPGTPGSRSRTPSLPTPPTREPKKVAVVRTPPKSPSSAKSRLQTAPVPMPDLKNVKSKIGSTENLKHQPGGGKVQIVYKPVDLSKVTSKCGSLGNIHHKPGGGQVEVKSEKLDFKDRVQSKIGSLDNITHVPGGGNKKIE	[19]
hCyp	167	2.51	GPMCNPTVFFDIAVDGEPLGRVSFELFADKVPKTAENFRALSTGEKGFGYKGSSFHRIIPGFMSQGGDFTRHNGTGGKSIYGEKFEDENFILKHTGPGILSMANAGPNTNGSQFFISTAKTEFLDGKHVVFGKVKEGMNIVEAMERFGSRNGKTSKKITIADSGQLC	[8]
an16	185	4.44	MHHHHHHPGAPAQTPSSQYGAPAQTPSSQYGAPAQTPSSQYGAPAQTPSSQYGAPAQTPSSQYGAPAQTPSSQYGAPAQTPSSQYGAPAQTPSSQYGAPAQTPSSQYGAPAQTPSSQYGAPAQTPSSQYGAPAQTPSSQYGAPAQTPSSQYGAPAQTPSSQYGAPAQTPSSQYGAPAQTPSSQYGAPAQTPSSQYV	[3]

Supplementary Table 2. Sequences of the peptides in the ABS_test set.

Name	Len.	Sequence
P02338.0	29	MRSFDQGSTRAPARERCRRQRPEGRSAQR
Q2KXY0	33	MFDNASTRNNKRERGKRQGKQTRTQRHADRSQT
P02338.1	33	MRGRMRSFDQGSTRAPARERCRRQRPEGRSAQR
Q9EP54	27	MACYPVNIRARGLGKNMGMKSRGRGKG
P27205	34	AGSKSRSRSRSRSRSKSPAKSASPKSAASPRASR
P35422	35	PSPTRRSKSRSKSRSRSRSASAGKAAKRAKSKTAK
Q91185	32	MRRQASLPARRRRRVRRTRVVRRRRRVGRRRH
P02321.0	34	PRRRREASRPVRRRRRYRRSTAARRRRRVVRRRR
Q9PS27	34	PRRRRQASRPVRRRRRTRRSTAERRRRRVVRRRR
P08130	33	PRRRRETSRPIRRRRRARRAPIRRRRRVVRRRR
P02321.1	34	PRRRRQASRPVRRRRRYRRSTAARRRRRVVRRRR
P25327	34	PRRRRRSSRPVRRRRRYRRSTAARRRRRVVRRRR
P69006	27	ARRRRRSSRPQRRRRRRRHGRRRRGRR
P83215	24	RRRRRRRRHRRRRGRRGRRSRGRR
synthetic	34	RRRRRRRRRRRRRRRRRRRRRRRRRRRRRRRRRR

Supplementary Table 3. Evaluation of idpGAN and excluded volume simulations (EVS) for reproducing the ensembles of the ABS_test peptides.

**Method**	**MSE_c**	**MSE_d [nm^2]**	**aKLD_d**	**EMD-dRMSD [nm]**	**KLD_r**
EVS	13.48 ± 3.44	0.33 ± 0.16	0.49 ± 0.19	0.38 ± 0.06	1.83 ± 0.95
idpGAN	9.53 ± 2.03	0.04 ± 0.02	0.26 ± 0.02	0.22 ± 0.02	0.25 ± 0.13

Average values are reported along with standard errors for all the peptides in the set (n = 15). The scores were obtained by comparing ensembles of 10,000 randomly sampled conformations. Source data are provided as a Source Data file.

Supplementary Table 4. Architecture and hyper-parameters of the neural network modules.

Module	Neural network	Structure
FC_input	Generator	*Input → {LinearLayer(n_{in}=16, n_{out}=64) → LeakyReLU(0.2) → LinearLayer(n_{in}=64, n_{out}=64) → LeakyReLU(0.2) → LinearLayer(n_{in}=64, n_{out}=64)} → Output*.
update_MLP	Generator	*Input → {LinearLayer(n_{in}=64+32, n_{out}=128) → LeakyReLU(0.2) → LinearLayer(n_{in}=128, n_{out}=64)} → Output.*
FC_3D	Generator	*Input → {LinearLayer(n_{in}=64, n_{out}=64) → LeakyReLU(0.2) → LinearLayer(n_{in}=64, n_{out}=3)} → Output.*
MLP_discriminator	Discriminator for CG data	*Input → {LinearLayer(n_{in}=L*(L-1)/2+20*L, n_{out}=1024, sn=True) → LeakyReLU(0.2) → LinearLayer(n_{in}=1024, n_{out}=1024, sn=True) → LeakyReLU(0.2) → LinearLayer(n_{in}=1024, n_{out}=1, sn=True) → Sigmoid()} → Output.*
MLP_discriminator	Discriminator for all-atom explicit solvent data	*Input → {LinearLayer(n_{in}=L*(L-1)/2+(L-3), n_{out}=1024) → LeakyReLU(0.2) → LinearLayer(n_{in}=1024, n_{out}=1024) → LeakyReLU(0.2) → LinearLayer(n_{in}=1024, n_{out}=1)} → Output*.

The *sn* argument in the linear layers indicates the use of spectral normalization (see main text).

Supplementary Table 5. Hyper-parameters in generator network of idpGAN for CG and explicit solvent all-atom Cα trace modeling.

**Hyper-parameter name**	**CG data**	**All-atom data**
Transformer block embedding dimension (d)	64	128
Feed forward dimension in the updater module of transformer blocks	128	256
Number of transformer blocks (n_t)	8	16
Number of attention heads (n_h)	8	12
Dimension of the query, key and values vectors	16	32
Maximum sequence separation in relative 2d positional embeddings	24	32

Supplementary Table 6. Parameters used for generating conformations with idpGAN during timing tests.

Protein length interval	Batch size
(0, 50]	4096
(50, 80]	2048
(80, 110]	1024
(110, 140]	512
(140, 200]	256

Supplementary Note 1

In this supplementary note, we describe the protocol used to run MCMC peptide simulations via CAMPARI[1].

Hamiltonian
Simulations were performed using the OPLS-AA/L force field and the ABSINTH implicit solvent model. The CAMPARI parameter set file *abs3.1_opls.prm* was used. Cutoffs for Lennard-Jones and electrostatic interactions were set at 10 and 14 Å respectively.

Peptide modeling
Peptides were capped with an acetyl group at the N-terminus and a N-methylamide group at the C-terminus. Histidine sidechains were protonated only at the ε position, making them neutrally charged.

System
Peptides were placed inside a spherical droplet. The radius of the droplet was set to 70 Å for peptides with 34 or less residues (that we defined as "short" peptides) and 100 Å for peptides with 35 or more residues (defined as "long" peptides). Na^+ and Cl^- ions were added to neutralize net peptide charges and to represent a 125 mM salt solution (resulting in 108 excess ion pairs for 70 Å droplets and 315 pairs for 100 Å droplets).

Sampling algorithm
Metropolis MCMC simulations were performed in the NVT ensemble at 298 K. The degrees of freedom in the simulations were backbone φ, ψ, ω torsion angles and sidechain χ torsion angles and rigid-body coordinates for peptides molecules and ions.

Monte Carlo move set
The move set was based on Mao et al.[1]

Simulations
For each peptide, 5 (for the training set peptides) or 20 (for the ABS_test peptides) independent MCMC simulations were performed using randomly generated initial conformations. For "short" peptides, we performed 1×10^6 equilibration and 2.5×10^7 production steps. For "long" peptides, we performed 2×10^6 equilibration and 5×10^7 production steps.

Output
Snapshots were saved every 5,000 steps during the production phase of the simulations, resulting in 5,000 snapshots for a "short" peptide simulation and 10,000 snapshots for a "long" peptide simulation.

Supplementary Note 2

In this supplementary note, we describe the details of the neural network for selecting the correct handedness of conformations generated by idpGAN.

Task
Binary classification task. Given the Cartesian coordinates of the Cα atoms of a peptide, the task is to classify the conformation as having the "correct" or "wrong" handedness.

Training set
We use the same dataset employed for training idpGAN to model Cα traces of all-atom peptides from ABSINTH simulations (see the main text).

Neural network
We adopt the exact same architecture of the idpGAN generator used to model conformations from ABSINTH simulations, except for the output module (see below).

Input
The input data is comprised by two parts: a sequence **t** containing structural information of a conformation and a sequence **a** containing amino acid information. For a peptide with L residues, **t** is a sequence of dimensions (L, 3). The first two channels contain the cosine and sine values of the L-3 torsion angles between all groups of four consecutive Cα atoms of the peptide (the first position and last two positions are padded with zeros). The third channel contains a mask with a value of 0 for the three positions originated from padding, and 1 for the L-3 remaining positions. **t** is fed to the network in the same way of the **z** sequence of the G network of idpGAN (see main text). The other part of the input is the amino acid encoding **a**, which is obtained and used by the selector network like the **a** encoding of the generator network of idpGAN (see main text).

Output
The last transformer block of the network outputs a sequence of dimensions (L, d) (with d = 96) which is summed along the length axis to obtain a d-sized vector. This vector is fed to the output module of the network, which is a fully-connected module with two linear layers with input dimension of d and a LeakyReLU activation. The module returns via a sigmoid activation a scalar value from 0 to 1, which corresponds to the probability of the input conformation to be of the "correct" handedness.

Training protocol
For training, we use batches containing the Cα Cartesian coordinates of peptides having the same length. The coordinates of the elements in a batch are reflected with a probability of 0.5, resulting in batches containing approximately half of conformations with the "correct" (original) handedness and half with the "wrong" (reflected) one. Additionally, Gaussian noise with σ = 0.5 Å is added to the coordinates to make sure that the network can also work with conformations generated by idpGAN (which may contain small inaccuracies). The training objective is binary cross-entropy for classifying the type of handedness. 1,750 conformations are randomly selected for each peptide during each training epoch and a batch size of 192 is used. For optimization, we use Adam (with β_1 = 0.9 and β_2 = 0.99) and adopt a learning rate of 0.0005. The training of a selector model lasts for 50 epochs.

Test set and performance
For testing the model, we use conformations from the peptides of the ABS_test set (see the main text). On a test set containing 1,750 conformations (half of them reflected, with no Gaussian noise added) from each peptide, the network obtains an accuracy score of 99.0%. This indicates that the input features used in this task contain the necessary information to classify the correct handedness of Cα traces.

Supplementary References

1 Mao, A. H., Crick, S. L., Vitalis, A., Chicoine, C. L. & Pappu, R. V. Net charge per residue modulates conformational ensembles of intrinsically disordered proteins. *Proc. Natl. Acad. Sci. U.S.A.* **107**, 8183-8188 (2010).

2 Cragnell, C., Durand, D., Cabane, B. & Skepo, M. Coarse-grained modeling of the intrinsically disordered protein Histatin 5 in solution: Monte Carlo simulations in combination with SAXS. *Proteins* **84**, 777-791 (2016).

3 Kohn, J. E. *et al.* Random-coil behavior and the dimensions of chemically unfolded proteins. *Proc. Natl. Acad. Sci. U.S.A.* **101**, 12491-12496 (2004).

4 Fuertes, G. *et al.* Decoupling of size and shape fluctuations in heteropolymeric sequences reconciles discrepancies in SAXS vs. FRET measurements. *Proc. Natl. Acad. Sci. U.S.A.* **114**, E6342-E6351 (2017).

5 Müller-Späth, S. *et al.* From the Cover: Charge interactions can dominate the dimensions of intrinsically disordered proteins. *Proc. Natl. Acad. Sci. U.S.A.* **107**, 14609-14614 (2010).

6 Smith, C. K. *et al.* Surface point mutations that significantly alter the structure and stability of a protein's denatured state. *Protein Sci.* **5**, 2009-2019 (1996).

7 Choy, W. Y. *et al.* Distribution of molecular size within an unfolded state ensemble using small-angle X-ray scattering and pulse field gradient NMR techniques. *J. Mol. Biol.* **316**, 101-112 (2002).

8 Hofmann, H. *et al.* Polymer scaling laws of unfolded and intrinsically disordered proteins quantified with single-molecule spectroscopy. *Proc. Natl. Acad. Sci. U.S.A.* **109**, 16155-16160 (2012).

9 Sherman, E. & Haran, G. Coil-globule transition in the denatured state of a small protein. *Proc. Natl. Acad. Sci. U.S.A.* **103**, 11539-11543 (2006).

10 Kjaergaard, M. *et al.* Temperature-dependent structural changes in intrinsically disordered proteins: formation of alpha-helices or loss of polyproline II? *Protein Sci.* **19**, 1555-1564 (2010).

11 Arbesu, M. *et al.* The unique domain forms a fuzzy intramolecular complex in src family kinases. *Structure* **25**, 630-640 e634 (2017).

12 Mittag, T. *et al.* Structure/function implications in a dynamic complex of the intrinsically disordered Sic1 with the Cdc4 subunit of an SCF ubiquitin ligase. *Structure* **18**, 494-506 (2010).

13 Wells, M. *et al.* Structure of tumor suppressor p53 and its intrinsically disordered N-terminal transactivation domain. *Proc. Natl. Acad. Sci. U.S.A.* **105**, 5762-5767 (2008).

14 Metrick, C. M., Koenigsberg, A. L. & Heldwein, E. E. Conserved Outer Tegument Component UL11 from Herpes Simplex Virus 1 Is an Intrinsically Disordered, RNA-Binding Protein. *mBio* **11** (2020).

15 Lens, Z. *et al.* Solution structure of the N-terminal transactivation domain of ERM modified by SUMO-1. *Biochem. Biophys. Res. Comm.* **399**, 104-110 (2010).

16 Riback, J. A. *et al.* Innovative scattering analysis shows that hydrophobic disordered proteins are expanded in water. *Science* **358**, 238-241 (2017).

17 Flanagan, J. M., Kataoka, M., Shortle, D. & Engelman, D. M. Truncated staphylococcal nuclease is compact but disordered. *Proc. Natl. Acad. Sci. U.S.A.* **89**, 748-752 (1992).

18 Nath, A. *et al.* The conformational ensembles of alpha-synuclein and tau: combining single-molecule FRET and simulations. *Biophys. J.* **103**, 1940-1949 (2012).

19 Mylonas, E. *et al.* Domain conformation of tau protein studied by solution small-angle X-ray scattering. *Biochemistry* **47**, 10345-10353 (2008).
