# Supplementary material for: Direct generation of protein conformational ensembles via machine learning
Source: Nat Commun. 2023 Feb 11;14:774. doi: 10.1038/s41467-023-36443-x (PMC9922302; doi:10.1038/s41467-023-36443-x)
Supplement: Supplementary file 3 — Reporting Summary [file 41467_2023_36443_MOESM3_ESM.pdf]

nature portfolio

Corresponding author(s): Michael Feig

Last updated by author(s): January 16, 2023

Reporting Summary

Nature Portfolio wishes to improve the reproducibility of the work that we publish. This form provides structure for consistency and transparency in reporting. For further information on Nature Portfolio policies, see our Editorial Policies and the Editorial Policy Checklist.

Statistics

For all statistical analyses, confirm that the following items are present in the figure legend, table legend, main text, or Methods section.

n/a	Confirmed	
☐	☒	The exact sample size (*n*) for each experimental group/condition, given as a discrete number and unit of measurement
☒	☐	A statement on whether measurements were taken from distinct samples or whether the same sample was measured repeatedly
☒	☐	The statistical test(s) used AND whether they are one- or two-sided<br>*Only common tests should be described solely by name; describe more complex techniques in the Methods section.*
☒	☐	A description of all covariates tested
☒	☐	A description of any assumptions or corrections, such as tests of normality and adjustment for multiple comparisons
☐	☒	A full description of the statistical parameters including central tendency (e.g. means) or other basic estimates (e.g. regression coefficient) AND variation (e.g. standard deviation) or associated estimates of uncertainty (e.g. confidence intervals)
☒	☐	For null hypothesis testing, the test statistic (e.g. *F*, *t*, *r*) with confidence intervals, effect sizes, degrees of freedom and *P* value noted<br>*Give P values as exact values whenever suitable.*
☒	☐	For Bayesian analysis, information on the choice of priors and Markov chain Monte Carlo settings
☒	☐	For hierarchical and complex designs, identification of the appropriate level for tests and full reporting of outcomes
☒	☐	Estimates of effect sizes (e.g. Cohen's *d*, Pearson's *r*), indicating how they were calculated

Our web collection on statistics for biologists contains articles on many of the points above.

Software and code

Policy information about availability of computer code

Data collection	Custom Python (3.8.10) code using the OpenMM (7.7.0) library was used to collect the molecular dynamics data for this study. Input files for the molecular dynamics simulations were obtained with the MMTSB Tool Set (v1) and CHARMM (v44b2) package. Markov chain Monte Carlo simulations were performed using the CAMPARI (4.0) package. Custom Python code using PyTorch (1.7.1) was used to obtain the generated datasets via a generative model. Custom code is available on github: https://github.com/feiglab/idpgan
Data analysis	All data was analysed using custom Python code using the MDTraj (1.9.5) and NGLview (3.0.1) libraries.

For manuscripts utilizing custom algorithms or software that are central to the research but not yet described in published literature, software must be made available to editors and reviewers. We strongly encourage code deposition in a community repository (e.g. GitHub). See the Nature Portfolio guidelines for submitting code & software for further information.

Data

Policy information about availability of data

All manuscripts must include a data availability statement. This statement should provide the following information, where applicable:

- Accession codes, unique identifiers, or web links for publicly available datasets
- A description of any restrictions on data availability
- For clinical datasets or third party data, please ensure that the statement adheres to our policy

The training set sequences were obtained from DisProt version 2021_06 available at https://disprot.org/download. Training set and IDP_test, ABS_test sequences,

and HB_val validation splits are available at https://github.com/feiglab/idpgan. Datasets analyzed and generated in the current study are available from the corresponding author on reasonable request. Raw data presented in figures and tables in the main text and Supplementary Information are provided in the Source Data file.

Human research participants

Policy information about studies involving human research participants and Sex and Gender in Research.

Reporting on sex and gender	N/A
Population characteristics	N/A
Recruitment	N/A
Ethics oversight	N/A

Note that full information on the approval of the study protocol must also be provided in the manuscript.

Field-specific reporting

Please select the one below that is the best fit for your research. If you are not sure, read the appropriate sections before making your selection.

☒ Life sciences ☐ Behavioural & social sciences ☐ Ecological, evolutionary & environmental sciences

For a reference copy of the document with all sections, see nature.com/documents/nr-reporting-summary-flat.pdf

Life sciences study design

All studies must disclose on these points even when the disclosure is negative.

Sample size	The sample size applies to the systems used for training machine learning. We considered all available disordered regions from DisProt with 20-200 residues.
Data exclusions	In the training data set, we excluded systems with redundant sequences (based on high sequence similarity) and systems with more than 200 residues to match computational resources and the complexity of the machine learning framework.
Replication	We carried out 10 independent training runs for the machine learning models, all of which resulted in similar models. Therefore, attempts at replication were successful.
Randomization	Our study is purely computational and we did not use experimental groups, therefore no random allocation scheme into experimental groups was applied.
Blinding	No blinding was applied. All of the analysis was carried out automatically via computer programs.

Reporting for specific materials, systems and methods

We require information from authors about some types of materials, experimental systems and methods used in many studies. Here, indicate whether each material, system or method listed is relevant to your study. If you are not sure if a list item applies to your research, read the appropriate section before selecting a response.

Materials & experimental systems

n/a	Involved in the study	
☒	☐	Antibodies
☒	☐	Eukaryotic cell lines
☒	☐	Palaeontology and archaeology
☒	☐	Animals and other organisms
☒	☐	Clinical data
☒	☐	Dual use research of concern

Methods

n/a	Involved in the study	
☒	☐	ChIP-seq
☒	☐	Flow cytometry
☒	☐	MRI-based neuroimaging
